# SIRPG expression positively associates with an inflamed tumor microenvironment and response to PD-1 blockade

**DOI:** 10.1007/s00262-024-03737-y

**Published:** 2024-06-04

**Authors:** Libo Luo, Minlin Jiang, Hong Wu, Yiqiang Liu, Haowei Wang, Caicun Zhou, Shengxiang Ren, Xiaoxia Chen, Tao Jiang, Chuan Xu

**Affiliations:** 1 Department of Medical Oncology, Shanghai Pulmonary Hospital and Thoracic Cancer Institute, Tongji University School of Medicine, No. 507 Zhengmin Road, Shanghai, 200433 China; 2 Department of Oncology and Cancer Institute, Department of Laboratory Medicine and Sichuan Provincial Key Laboratory for Human Disease Gene Study, Sichuan Academy of Medical Sciences, Sichuan Provincial People’s Hospital, University of Electronic Science and Technology of China, No. 32 1st Ring Road, Chengdu, 610072 China

**Keywords:** Programmed cell death 1 receptor, PD-1, SIRPG, Tumor microenvironment, Biomarker, Lung cancer

## Abstract

**Background:**

This study aimed to investigate the relationship between signal regulatory protein gamma (SIRPG) and tumor immune microenvironment phenotypes or T cell mediated-adaptive antitumor immunity, and its predictive value for response to PD-1 blockade in cancers.

**Methods:**

Pan-cancer analysis of SIRPG expression and immune deconvolution was performed using transcriptomic data across 33 tumor types. Transcriptomic and clinical data from 157 patients with non-small-cell lung cancer (NSCLC) and melanoma received PD-1 blockade were analyzed. Expression characteristics of SIRPG were investigated using single-cell RNA sequencing (scRNA-seq) data of 103,599 cells. The effect of SIRPG expression was evaluated via SIRPG knockdown or overexpression in Jurkat T cells.

**Results:**

The results showed that most cancers with high SIRPG expression had significantly higher abundance of T cells, B cells, NK cells, M1 macrophages and cytotoxic lymphocytes and increased expression level of immunomodulatory factors regulating immune cell recruitment, antigen presentation, T cell activation and cytotoxicity, but markedly lower abundance of neutrophils, M2 macrophages, and myeloid-derived suppressor cells. High SIRPG expression was associated with favorable response to PD-1 blockade in both NSCLC and melanoma. scRNA-seq data suggested SIRPG was mainly expressed in CD8^+^ exhausted T and CD4^+^ regulatory T cells, and positively associated with immune checkpoint expression including PDCD1 and CTLA4. In vitro test showed SIRPG expression in T cells could facilitate expression of PDCD1 and CTLA4.

**Conclusion:**

High SIRPG expression is associated with an inflamed immune phenotype in cancers and favorable response to PD-1 blockade, suggesting it would be a promising predictive biomarker for PD-1 blockade and novel immunotherapeutic target.

**Supplementary Information:**

The online version contains supplementary material available at 10.1007/s00262-024-03737-y.

## Introduction

Immunotherapy, targeting the interactions of PD-1 and PD-L1, has revolutionized the treatment landscape and significantly improved the overall survival (OS) of patients with various cancers including non-small-cell lung cancer (NSCLC), melanoma, breast, renal, gastrointestinal, head and neck cancer, and so on [1–6]. Despite of the huge success of immunotherapy, there are several mutual dilemmas that most cancer patients would inevitably encounter [7–10], including but not limited to (i) low response rate: the average objective response rate of PD-1 blockade monotherapy was approximately 20% in biomarker-unselected populations across distinct solid tumors; (ii) relatively short progression-free survival (PFS): for example, the median PFS was 3–6 months in patients with advanced NSCLC receiving second or later line anti-PD-1/PD-L1 monotherapy [11–14]; (iii) long-term survival benefit in only a tiny proportion of populations. Thus, developing robust and reliable predicting biomarkers, rational combination therapeutic strategies, and novel immunotherapeutic targets, is the key for the future of immunotherapy [15–19].

The rational combination of novel immunotherapeutic drugs and PD-1 blockade has been recently demonstrated to markedly improve the treatment outcomes compared to PD-1 blockade alone in various solid tumors. In a phase II–III, global, double-blind, randomized trial, patients with previously untreated metastatic or unresectable melanoma receiving relatlimab (a LAG-3-blocking antibody) plus nivolumab (a PD-1-blocking antibody) had dramatically longer PFS than those receiving nivolumab alone (median PFS 10.1 vs. 4.6 months, *P* = 0.006) [20]. Similarly, a randomized, double-blinded, placebo-controlled phase II trial reported that tiragolumab (anti-TIGIT antibody) plus atezolizumab (anti-PD-L1 antibody) significantly improved objective response rate (ORR), PFS and OS in patients with newly diagnosed metastatic PD-L1-positive NSCLC compared with placebo plus atezolizumab [21]. These findings suggest that the identification of novel immunotherapeutic targets and development of their agonists/antagonists could help expand the benefit populations and extend the survival benefit of current immunotherapies.

Our previous study unraveled a novel immune inhibitory function of signal regulatory protein gamma (SIRPG), a transmembrane glycoprotein with extracellular immunoglobulin-like domains that belongs to the signal regulatory protein family [22]. We found that high SIRPG expression in lung cancer cells mediates their cancer stem-like cell properties. Additionally it facilitates the dephosphorylation of MST1 by PP2A to activate the Hippo/YAP signaling cascade, which subsequently upregulates the expression of CD47 and leads to immune escape [22]. Blockade of SIRPG using its monoclonal antibody could significantly suppress tumor growth in vivo [22]. In addition, previous studies revealed that SIRPG is preferentially expressed in T cells and activated natural killer (NK) cells [23]. SIRPG expression in T cells could positively regulate the activation of T cells in the situation of chronic stimulation via interaction with CD47 [24]. These findings indicate that SIRPG plays a significant role in immune response and would be a novel immunotherapeutic target. However, the associations between SIRPG expression and tumor immune microenvironment phenotypes or T cell mediated-adaptive antitumor immunity, and its predictive value for PD-1 blockade remain largely unknown.

Here, we conducted the integrated immunogenomic analysis of clinical specimens from The Cancer Genome Atlas (TCGA) and Gene Expression Omnibus (GEO) datasets across various cancer types and 157 cancer patients who received PD-1 blockade with available transcriptomic data and treatment outcomes. Expression characteristics of SIRPG in single cells were investigated using high-quality single-cell RNA sequencing (scRNA-seq) data from 27 lung cancer samples. The current results suggest that high SIRPG expression was highly associated with an inflamed tumor immune microenvironment and favorable response to PD-1 blockade in NSCLC and melanoma. Additionally, we found that SIRPG expression in T cells could regulate the expression of several immune-related molecules including PD-1 and CTLA-4, resulting in the transition of T cell’s phenotype and cytotoxicity. Based upon these findings, we propose that SIRPG expression could serve as a promising biomarker to predict response to PD-1 blockade and the antitumor effect of blockade of both SIRPG and PD-1 warrants further investigation.

## Materials and methods

### TCGA datasets and analysis

The genomic and transcriptomic data generated by TCGA Program on 33 tumor types were downloaded from the cBioPortal online database (https://www.cbioportal.org) [25]. The expression level of SIRPG of normal, primary and/or metastatic tumor tissues and the Pearson correlation analysis between SIRPG expression and immune cells abundance, immune-related markers expression [including major histocompatibility complex (MHC), immunostimulatory, immunoinhibitory and cytotoxic molecules] were estimated using a web server, named TIMER (Tumor Immune Estimation Resource, https://cistrome.shinyapps.io/timer/) [26]. Kaplan–Meier curves with log-rank tests were used to determine the survival differences between tumors with high and low SIRPG expression via the GEPIA2 web server (version 2 of the Gene Expression Profiling Interactive Analysis; http://gepia2.cancer-pku.cn/#analysis).

### Immune deconvolution

The R packages of TIMER, MCP-counter, TIDE, EPIC, CIBERSORT, CIBERSORTx, QUANTISEQ and XCELL were applied to infer the absolute abundance of major immune cell types [including B cells, CD4^+^ T cells, CD8^+^ T cells, NK cells, M1/M2 macrophages, myeloid-derived suppressor cells (MDSCs), monocytic lineage, dendritic cells (DCs), neutrophils, endothelial cells, fibroblasts, and related subsets] [26–32]. The deconvolution results were compared between high and low SIRPG expression groups using Student's *t* test. Benjamini–Hochberg method was also leveraged to correct the *P* values and the false discovery rates (FDR, *q* values) were calculated.

### Correlation analysis between SIRPG expression and TMB/MSI levels

The “maftools” R package was applied to analyze the mutational data. MSI score was obtained from the TCGA database. Correlation between SIRPG expression and TMB or MSI was conducted using Spearman correlation analysis. Radar plots were applied to map the correlations.

### Differential gene expression analysis

R package “edgeR” was utilized to determine differentially expressed genes (DEGs) between high and low SIRPG expression groups. Cutoffs of log_2_ (fold-change) > 1.5 or <  − 1.5 and FDR < 0.05 were applied to select the most significant DEGs. We listed all of the DEGs by using volcano plot and the top 25 upregulated genes in high versus low SIRPG expression group in the form of heatmap.

### Pathway enrichment analysis

We applied three methods (GO, KEGG, GSEA) to perform the pathway enrichment analysis. The curated gene sets of reported signaling pathways (from the KEGG, Hallmark, PID, Reactome databases) were downloaded from the Molecular Signature Database (http://software.broadinstitute.org/gsea/msigdb/index.jsp). R package “clusterProfiler” was used for GO term analysis, and GSEA software V.4.1.0 was utilized to study the relevant pathways between high and low SIRPG expression groups.

### Public datasets of anti-PD-1/PD-L1 treated cohort

A total of four public datasets were downloaded from previous studies. The clinical and bulk RNA-seq data of 43 NSCLC patients received PD-1 blockade monotherapy, along with response data available, were downloaded from Jung et al. [33] and Cho et al. [34]. For validation, we also downloaded the response and bulk RNA-seq data of 114 melanoma patients treated with anti-PD-1/PD-L1 monotherapy from Van Allen et al. [35] and Gide et al. [36]. The clinical responses were assessed by the Response Evaluation Criteria in Solid Tumors version 1.1 guideline (RECIST v1.1), including complete response (CR), partial response (PR), stable disease (SD) or progressive disease (PD). Responders were defined as patients received PD-1 blockade with CR or PR. Non-responders were defined as patients received PD-1 blockade with SD or PD. The definition of PFS and OS was consistent with their corresponding published studies. Kaplan–Meier curves with two-sided log-rank tests and Cox proportional hazards model with calculated hazard ratios (HRs) and 95% confidence intervals (CIs) were used to determine the survival difference.

### Cell culture

Jurkat T cells (Clone E6-1, ATCC, Germany) were cultured in RPMI-1640 medium (Gibco) supplemented with 10% fetal bovine serum (FBS) and 1% penicillin/streptomycin (#15140-122 Gibco, CA). Phorbol myristate acetate (PMA; absin, Shanghai) and anti-CD3 (1 μg/mL, OKT3; Biolegend, CA) were used to activate T cells at concentrations of 100 ng/mL and 1 μg/mL, respectively. Cells were typically stimulated with PMA/ anti-CD3 for 6 h, unless stated otherwise.

### Virus production

Full-length human SIRPG (NM_080816) cloned into the pLVX-CMV-EGFP-3FLAG vector were purchased from SunBio (Shanghai, China) for the overexpression assay. The short hairpin RNA (shRNA) specifically targeting SIRPG cloned into pMAGic4.1-U6 promoter-puro-GFP-shRNA vector was purchased from SunBio (Shanghai, China) for the knockdown assay. The sequences were listed as follows: shSIRPG#1 forward: 5′-CCGGGCTCCTGTTGGTCACAGTTCTCAAGAGAAACTGTGACCAACAGGAGCTTTTTTG-3′, reverse: 5′-AATTCAAAAAAGCTCCTGTTGGTCACAGTTTCTCTTGAGAACTGTGACCAACAGGAGC-3′; shSIRPG#2 forward: 5′-CCGGGCCGGGAATTAATCTACAATTCAAGAGATTGTAGATTAATTCCCGGCTTTTTTG-3′, reverse: 5′-AATTCAAAAAAGCCGGGAATTAATCTACAATCTCTTGAATTGTAGATTAATTCCCGGC-3′. The primers used to amplify the full-length sequence of human SIRPG were as follows: forward: 5′-GAGGAAGTCGGTGAAGAACGG-3′, reverse: 5′-CTGAGCGGGGTTCATGTAGG-3′.

### Target cell transduction

Jurkat T cells were cultured in RPMI-1640 with 10% FBS and 1% penicillin/streptomycin. For infection, 1.5 × 10^6^ cells were cultured in 2 mL complete medium mixed with virus (MOI = 10) into a well of a 6-well plate, and incubated at 37 °C. 48 h post infection, the medium was changed to complete medium with 4 μg/mL puromycin (BD Biosciences, USA). Selection was done for 48 h. Six days after infection, the overexpression or knockdown efficiency was detected by quantitative real-time polymerase chain reaction (qRT-PCR) and western blot.

### Western blot

Jurkat T cells were resuspended in cell lysis buffer containing 1 mM PMSF (ST507, Beyotime, Shanghai) to extract the total protein. The samples were separated by SDS-PAGE (PG112, Epizyme, Shanghai), and proteins were transferred to Polyvinylidene Difluoride membranes (Millipore, IPVH00010, MA, United States). Antibodies used were the following: SIRPG (sc53112, Santa Cruz, CA), PD-1 (BE0146, Bioxcell, NH), GAPDH (#5174, CST, Mass). We used horse radish peroxidase (HRP) labeled Goat-anti Rabbit (A0208, Beyotime, Shanghai), HRP labeled Goat-anti Mouse (A0216, Beyotime, shanghai) as secondary antibodies. Positive bands were visualized by enhanced chemiluminescence (Millipore, WBKLS0100, MA, United States).

### qRT-PCR

mRNA was extracted from cells using RNA extraction kit (220,011, fastagen, Shanghai), and qRT-PCR was performed using a SYBR Prime Script RT-PCR kit (RR820, TaKaRa, Japan) in a CFX Connect Real-Time System (Bio-Rad, CA, United States). GAPDH was used as the internal control. The PCR conditions were set as follows: denaturation at 95 °C for 30 s, followed by amplification for 45 cycles and quantification (95 °C for 5 s, 60 °C for 30 s), and melting curve (65–95 °C with 0.5 °C increment each cycle). Each sample was tested in triplicates. The primer sequences were listed as follows (Supplemental Table S1): β-actin: forward: 5′-TCTCCCAAGTCCACACAGG-3′, reverse: 5′-GGCACGAAGGCTCATCA-3′; interferon-γ (IFNγ): forward: 5′-AGCTCTGCATCGTTTTGGGTT-3′, reverse: 5′-GTTCCATTATCCGCTACATCTGAA-3′; PDCD1: forward: 5′-CAGTTCCAAACCCTGGTGGT-3′, reverse: 5′-GGCTCCTATTGTCCCTCGTG-3′; SIRPG: forward: 5′-TCCTCCTGGTCCTTTCCT-3′, reverse: 5′-GGCTGTCTTTCCAACTGTG-3′; GZMK: forward: 5′-CGTTTGTGGAGGTGTTCTG-3′, reverse: 5′-GAGAGAGTGTGCGCCTAAA-3′; CTLA-4: forward: 5′-GCAGTTAGTTCGGGGTTG-3′, reverse: 5′-CATTCTGGCTCTGTTGGG-3′.

### Flow cytometry analysis

The harvested Jurkat T cells were stained with fluorochrome-conjugated antibodies in the darkness under 4 °C for 30 min: Human anti-CD45-APC Cy7 (BD, Catalog: 304014, NJ), Human anti-IFNγ-PE (BD, Catalog: 562016, NJ), and then analyzed by flow cytometry. We used FlowJo v.10 software for data analysis.

### Enzyme-linked immunosorbent assay (ELISA)

1 × 10^5^ activated Jurkat T cells were incubated in 96 wells with 200 μL RPMI-1640 complete medium for 24 h. The levels of IFNγ in conditioned media were detected using ELISA kit in accordance with the manufacturer’s instructions (RK00015, Abclonal, Wuhan). The levels of GZMK from cultured media were detected by ELISA kit (ab314368, Abcam, Cambridge). The relative absorbance at 450 nm was measured using an automated ELISA reader (Bio-Rad model 550, Irvine, CA). The concentration was determined by comparing optical density value with standard curve.

### scRNA-seq dataset

Two scRNA-seq datasets of Zhuang et al. (GSE150938 dataset, https://www.ncbi.nlm.nih.gov/geo/query/acc.cgi?acc=GSE150938) as well as Ahn et al. (GSE131907 dataset [37], https://www.ncbi.nlm.nih.gov/geo/query/acc.cgi) were collected from GEO database (https://www.ncbi.nlm.nih.gov/geo/info/). Data from 12 lung ground glass nodule (GGN) samples were obtained from GSE150938 dataset and 15 lung primary tumor samples were obtained from GSE131907 dataset.

### Data processing for scRNA-seq data

For each study, we obtained the processed scRNA-seq dataset and author-supplied annotations. Cell Ranger Single-Cell Software Suite (v3.0.0 and v2.1.0) based on the GRCh38 human reference genome was used to align and quantify the droplet-based sequencing raw data with default parameters. The alignment reference and software were both provided by 10 × Genomics (https://support.10xgenomics.com). The raw count data were analyzed using R 4.1.1 based on Seurat (v4.0.4) R package for downstream analysis. First, we applied quality control to the preliminary filtered data. Cells with < 500 UMIs and > 10% mitochondrial gene count were filtered out. We also removed the potential doublets by filtering cells with UMI count more than 60,000. After that, all genes were normalized and principal component analysis (PCA) was performed on the first 2000 highly variable genes and the resolution parameter to identify clusters was set to 0.5. Two nonlinear dimensional reduction methods, t-distributed stochastic neighbor embedding (tSNE) [38] and uniform manifold approximation and projection (UMAP) [39], were performed for further cell visualization. We labeled T helper cells as “Th”, effective memory T cells as “Tem”, central memory T cell as “Tcm”, tissue-resident memory T cells as “Trm”, effector T cells as “Teff”, T cell exhaustion as “Tex”.

### Integration of two scRNA-seq datasets based on harmony

Harmony (v0.1.0) R package applies principal component analysis to embed transcriptome expression spectra into low-dimensional spaces, and then applies an iterative process to remove dataset-specific effects (https://github.com/immunogenomics/harmony) [40]. Based on harmony, the batch effects of cells between the two datasets were removed.

### Correlation analysis in scRNA-seq data

The correlation analysis of expression of SIRPG and MHC, immunostimulatory, immunoinhibitory, and cytotoxic molecules in all T cells, CD8^+^ Tex cells and CD4^+^ Treg cells were conducted by Pearson test. Corrplot (v0.92) R package was utilized for plotting. “X” in the heatmap represents no statistical significance. We included all cells for analysis.

### Statistical analysis

Comparisons between high and low SIRPG expression groups were performed using Student’s *t* test and/or Wilcoxon signed-rank test. Mann–Whitney *U*-tests and/or Kruskal–Wallis rank-sum tests were leveraged for comparisons of continuous variables across groups. To determine differences in enriched pathways between groups, we used two-tailed Fisher’s exact tests with Benjamini–Hochberg correction for multiple hypothesis testing to generate *q* values [41]. Pearson correlation analysis were calculated to evaluate the relatedness of SIRPG expression and immune cells abundance, immune-related markers expression. All statistical significance testing was two-sided and *P* values or *q* values < 0.05 were considered statistically significant. All tests were performed with the R environment v4.0 or GraphPad Prism version 6.0.

## Results

### SIRPG is highly expressed in various cancers

**Fig. 1:**
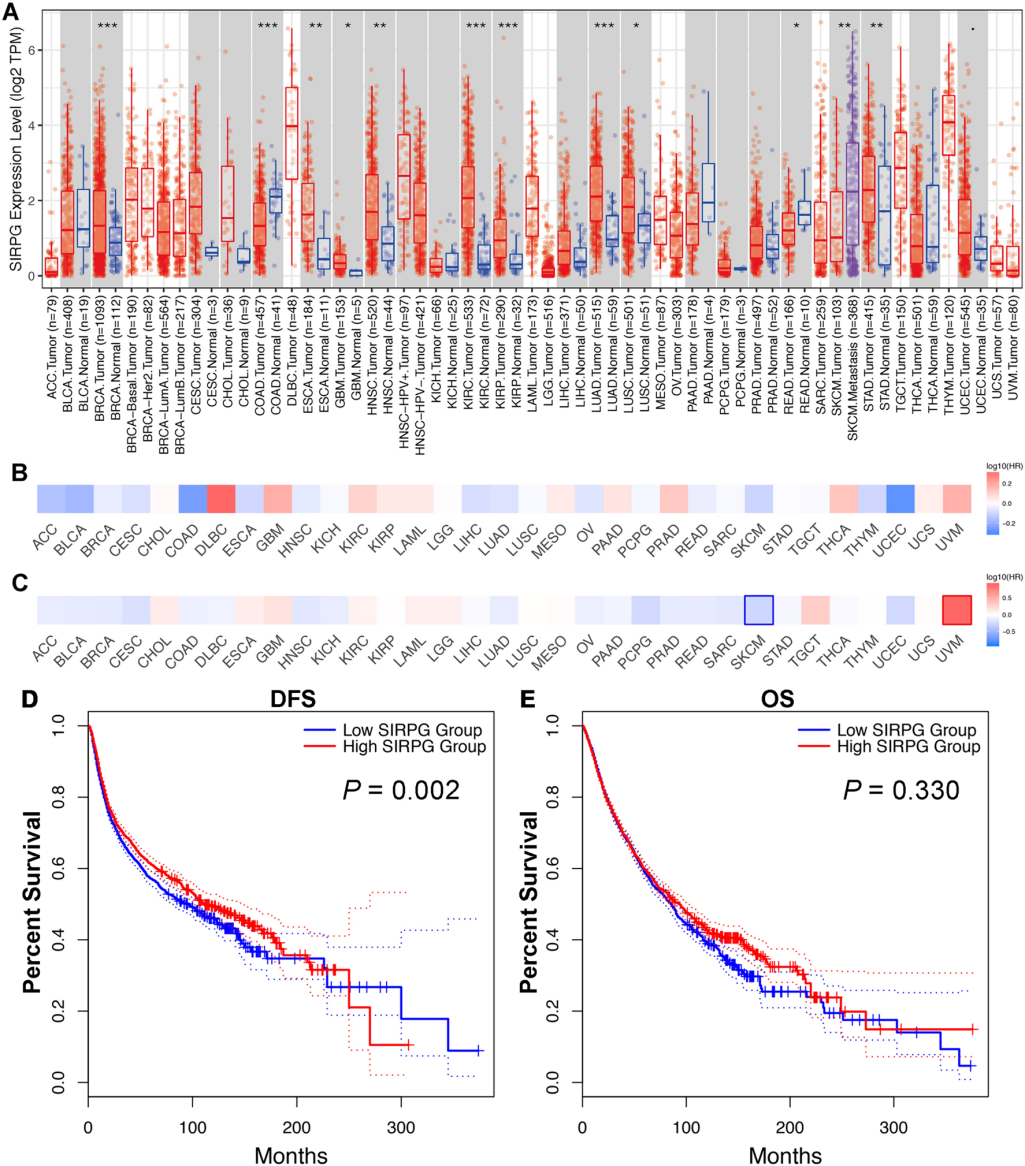
SIRPG is highly expressed in various cancers. **A** SIRPG expression based on TCGA database. Prognostic values of SIRPG expression for DFS (**B**) or OS (**C**). DFS (**D**) or OS (**E**) of high and low SIRPG expression groups. ·*P* < 0.1; *P < 0.05; **P < 0.01; ***P < 0.001. TPM, Transcripts per kilobase million; HR, Hazard ratio; DFS, Disease-free survival; OS, Overall survival

Firstly, we analyzed the distinct expression levels of SIRPG between normal and tumor tissues using transcriptomic datasets from the TCGA program. Among them, tumor tissues from 10 types [breast (BRCA), esophageal carcinoma (ESCA), glioblastoma multiforme (GBM), head and neck squamous cell carcinoma (HNSC), kidney renal clear cell carcinoma (KIRC), kidney renal papillary cell carcinoma (KIRP), lung adenocarcinoma (LUAD), lung squamous cell carcinoma (LUSC), stomach adenocarcinoma (STAD), uterine corpus endometrial carcinoma (UCEC)] showed consistently and significantly higher level of SIRPG expression than matched normal tissues, while colon adenocarcinoma (COAD) and rectum adenocarcinoma (READ) had markedly lower level of SIRPG expression than matched normal tissues (Fig. 1A). Metastatic tumors from skin cutaneous melanoma (SKCM) showed significantly higher level of SIRPG expression than their primary tumor tissues, suggesting a hypothetically biological role of SIRPG in SKCM distant metastasis (Fig. 1A). We then investigated the pan-cancer prognostic value of SIRPG expression in these cancers. We defined the high expression group as tumors with expression level ≥ median level and low expression group as that with expression level < median level in each cancer type. Consistently in multiple TCGA cancer cohorts, most cancer types with high SIRPG expression level had similar disease-free survival (DFS, Fig. 1B) and OS (Fig. 1C) after adjustment for potential confounding factors such as FDR. In SKCM, high SIRPG expression was associated with significantly longer OS whereas it was correlated with shorter OS in uveal melanoma (Fig. 1C). Overall, although tumors with high SIRPG expression had significantly better DFS than those with low expression level (*N* = 9476, *P* = 0.002; Fig. 1D), no statistical difference was observed in OS between two groups (*N* = 9476, *P* = 0.330; Fig. 1E).

### SIRPG confers “hot” tumor immune phenotypes in various cancers

**Fig. 2:**
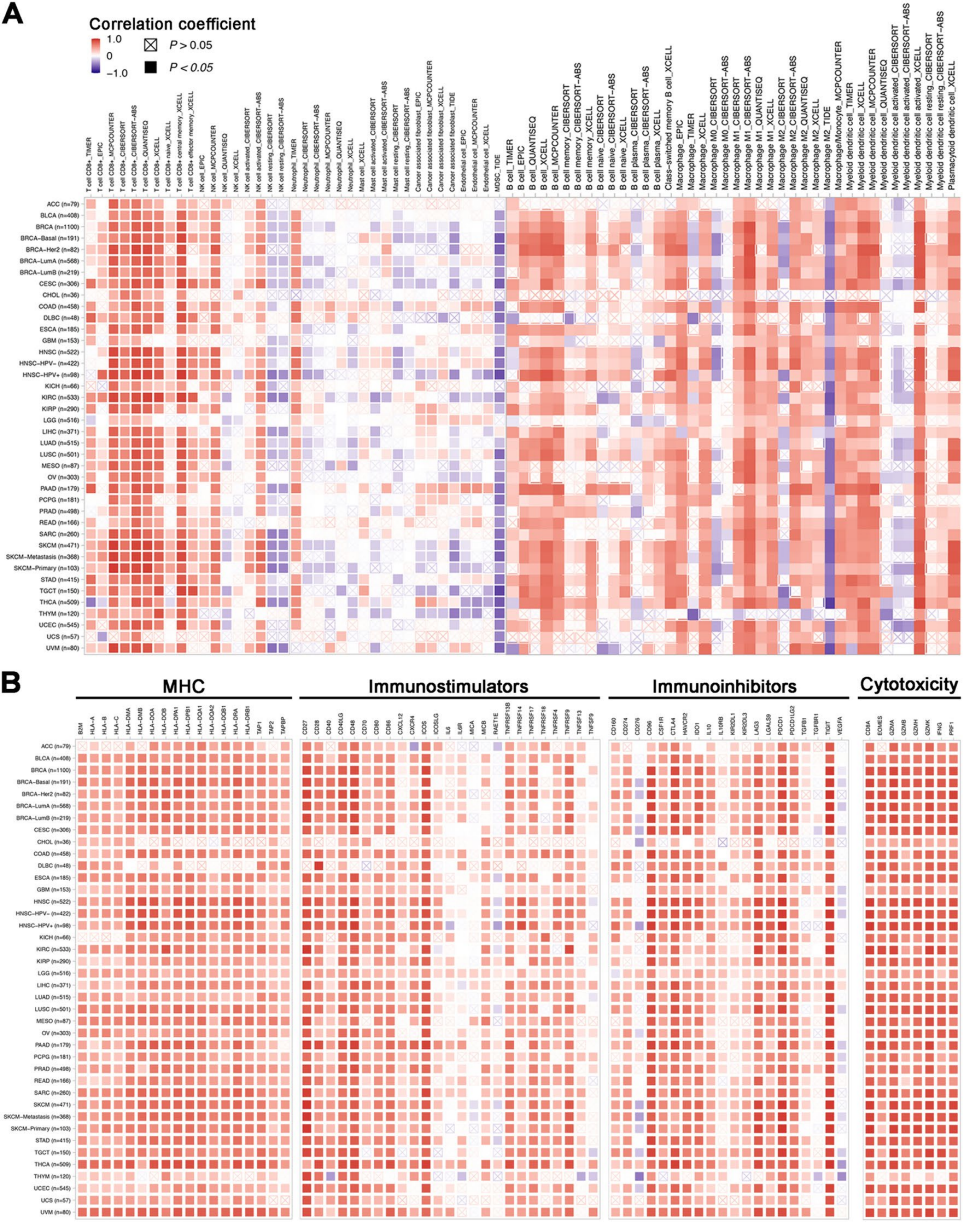
High SIRPG expression confers “hot” tumor immune phenotypes in cancers. **A** The correlation of SIRPG expression with distinct immune cellular subtypes among tumors. **B** The association between SIRPG expression and major histocompatibility complex, immunostimulatory, immunoinhibitory, and cytotoxic molecules

We next surveyed the relevance between SIRPG expression and immune cell abundance and cellular composition. We conducted the immune deconvolution analysis of the bulk RNA-seq data from TCGA by leveraging various methods including TIMER, MCP-counter, TIDE, EPIC, CIBERSORT, CIBERSORTx, QUANTISEQ and XCELL. Consistently across most cancer types with high SIRPG expression (vs. low SIRPG expression), we observed remarkable increase in abundance of T, B, NK, T follicular helper cells, and M1 macrophages, and significant decrease in abundance of neutrophils, M2 macrophages and MDSC (Fig. 2A). We further analyzed the T cell subsets including naïve, memory, central memory, effector memory, memory resting/activated, Th1, Th2 CD4^+^ T, and naïve, central memory, effector memory CD8^+^ T, and regulatory T cells (Tregs). As shown in Fig. S1, cancers with high SIRPG expression had significantly higher abundance of CD8^+^ T cells, especially central memory CD8^+^ T cells than those with low SIRPG expression in various cancers.

We then identified the relationship between SIRPG expression and the expression levels of MHC, immunostimulatory, immunoinhibitory, and cytotoxic molecules in tumor tissues based on bulk RNA-seq data. Interestingly, the expression levels of immune checkpoints including PDCD1 (PD-1), CTLA4, LAG3, TIGIT, HAVCR2 (TIM3) were positively correlated with SIRPG expression. Also, CD274 (PD-L1) showed positive association with SIRPG, whereas RATE1E, CD276 and VEGFA expression seemed to be negatively correlated with SIRPG expression in a subset of TCGA cancers (Fig. 2B). Besides, significantly positive correlations were observed between gene expression of SIRPG and MHC, immunostimulatory, and other immunoinhibitory molecules in most cancers (Fig. 2B). Several studies have revealed a direct or an indirect impact of VEGFA on the T cell-based immunosuppression. CD276 inhibits antigen-presenting cells (APCs) and stimulates Tregs which results in IL-2 suppression. Conversely, the expression of genes regulating T cell activation and cytotoxicity including CD8A, EOMES, GZMA, GZMB, GZMH, GZMK, IFNG and PRF1 were massively increased in SIRPG-expressed tumors across multiple cancer types (Fig. 2B). Collectively, upregulation of these immunomodulatory factors regulating immune cell recruitment, antigen presentation, T cell activation alongside with increased cytotoxicity can lead to “hot” immune phenotypes in tumors with high SIRPG expression.

We further assessed the associations between SIRPG expression and tumor mutational burden (TMB) or microsatellite instability (MSI) levels. We found that SIRPG expression was positively associated with TMB in COAD, KICH, and TGCT, while it showed a negative association with TMB in HNSC, LGG, LIHC, LUSC, and THCA (Fig. S2A). Regarding MSI, we observed a negative association between SIRPG expression and MSI in most cancer types, including ACC, BLCA, BRCA, CESC, CHOL, DLBC, ESCA, GBM, HNSC, KIRP, LIHC, LUAD, LUSC, MESO, OV, PAAD, PCPG, READ, SARC, SKCM, STAD, TGCT, and THYM (Fig. S2B). However, it was positively correlated with MSI in UCEC and UVM (Fig. S2B). The results along with our previous findings highlight the complex role of SIRPG in tumors and their microenvironment, warranting further investigation.

### NSCLC with high SIRPG expression showed immune-responsive phenotypes

**Fig. 3:**
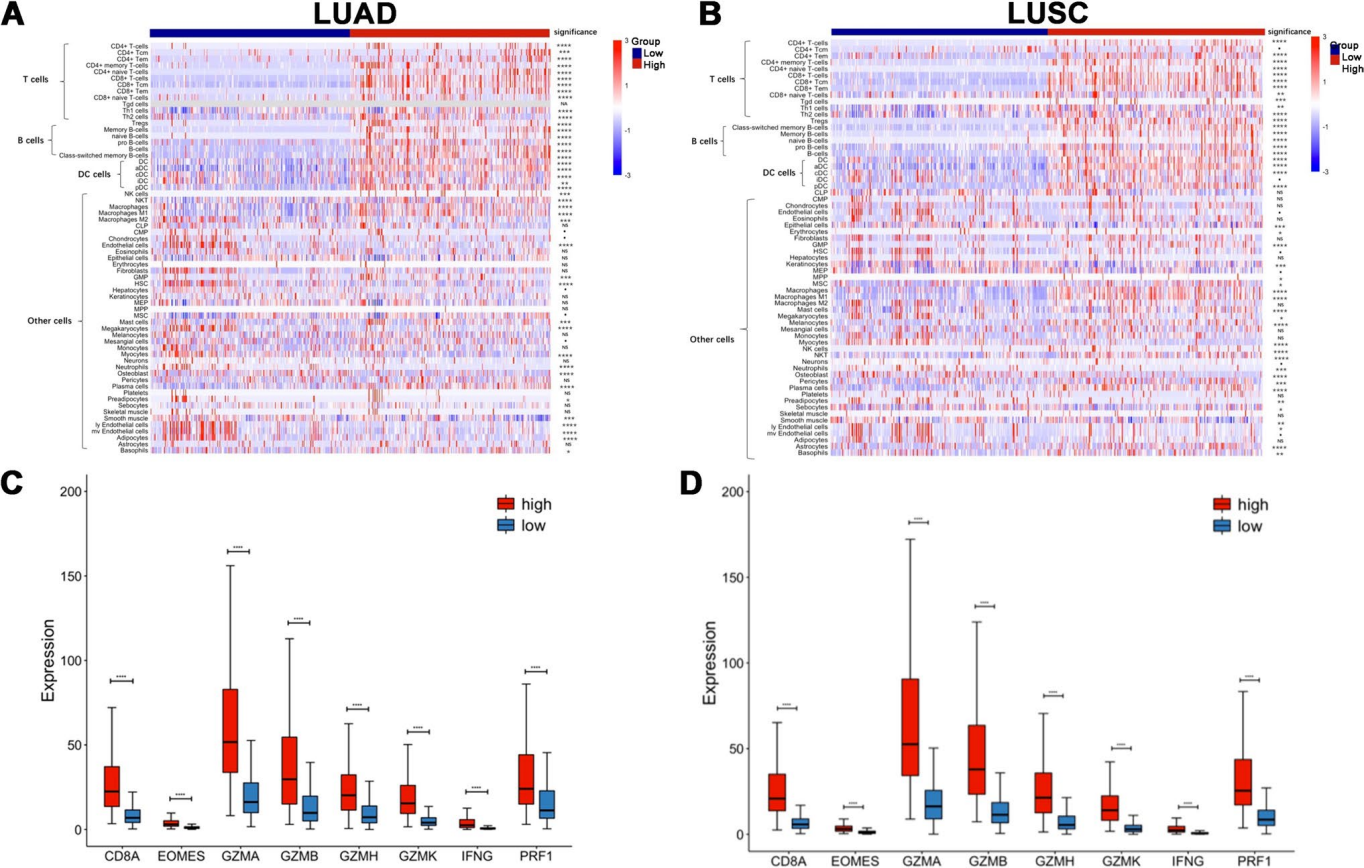
SIRPG^high^ NSCLC showed immune-responsive phenotypes. Immune landscapes of LUAD (**A**) or LUSC (**B**) according to SIRPG expression. Association of SIRPG expression and T cells related genes in LUAD (**C**) or LUSC (**D**). ****P < 0.0001. LUAD, Lung adenocarcinoma; LUSC, Lung squamous cell carcinoma

**Fig. 4:**
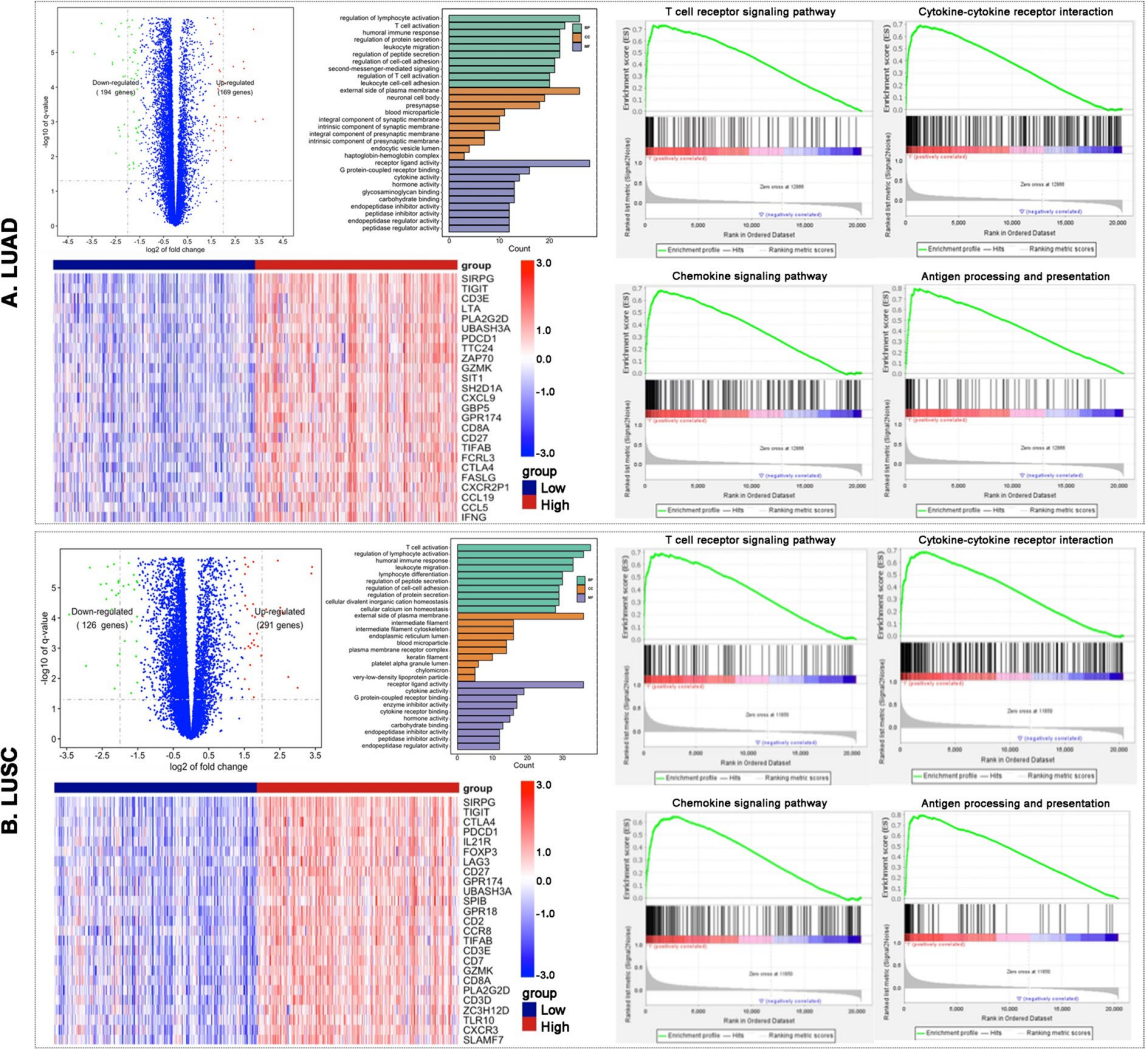
SIRPG expression related difference expression genes (DEGs) and biological pathways in NSCLC. DEGs, top 25 DEGs, top 30 enriched GO terms, and GSEA enriched pathways between high and low SIRPG expression groups in LUAD (**A**) or LUSC (**B**). LUAD, Lung adenocarcinoma; BP, Biological process; CC, Cellular component; MF, Molecular function; LUSC, Lung squamous cell carcinoma

Considering the high morbidity and mortality, we then emphatically compared immune cell abundance and cellular composition of NSCLC including LUAD and LUSC between the groups with high or low SIRPG expression. We firstly observed the expression level of SIRPG was analogous across different stages of both LUAD (Fig. S3A) and LUSC (Fig. S3B). In line with the above-mentioned results, we observed remarkable increase in the abundance of T cells, B cells, NK cells, DCs, M1 macrophages and other cytotoxic lymphocytes and decrease in abundance of neutrophils and Th17 cells revealed by XCELL, CIBERSORT and ImmuCellAI in both LUAD (Figs. 3A, S4A and S5) and LUSC cohort (Figs. 3B, S4B and S6). We then examined the distinct expression levels of MHC, immunostimulatory, immunoinhibitory and cytotoxic molecules between high and low SIRPG expression groups in two cohorts. Either LUAD or LUSC with high SIRPG expression was associated with significantly high expression levels of antigen presentation machinery [β2-microglobulin (B2M), HLA-A, HLA-B, HLA-C, HLA-DRB1, HLA-DRB5, TAP1, TAP2, TAPBP], immunostimulatory (CD28, CD40, CD80, CD86, ICOS), and immunoinhibitory (CD274, PDCD1, CTLA4, LAG3, TIGIT, HAVCR2) molecules (Figs. S7 and S8). Importantly, lung tumors with high SIRPG expression had dramatically high expression of genes regulating T cell activation and cytotoxicity including CD8A, EOMES, GZMA, GZMB, GZMH, GZMK, IFNG and PRF1 (Fig. 3C, D). To further survey the significant impact of SIRPG expression on tumor immune phenotypes, we compared the transcriptome characteristics between lung tumors with high and low SIRPG expression. In LUAD, there were 169 upregulated genes and 194 downregulated genes (Fig. 4A). In LUSC, 291 genes were upregulated and 126 genes were downregulated (Fig. 4B). Most of the top 25 upregulation genes in high versus low SIRPG expression group were the above-mentioned MHC, immunostimulatory, immunoinhibitory and cytotoxic molecules. Moreover, GSEA, GO, and KEGG pathway analyses revealed that lung tumors with high SIRPG expression were enriched in T cell receptor, T cell activation, cytokine–cytokine receptor interaction, chemokine and antigen processing and presentation signaling pathways (Figs. 4, S9 and S10). We also listed the top 10 enriched pathways in Fig. S11. Together these findings revealed that lung tumors with high SIRPG expression had inflamed immune phenotypes, indicating the significant role of SIRPG in cancer-immunity cycle of lung tumors.

### SIRPG expression was associated with response to PD-1 blockade

**Fig. 5:**
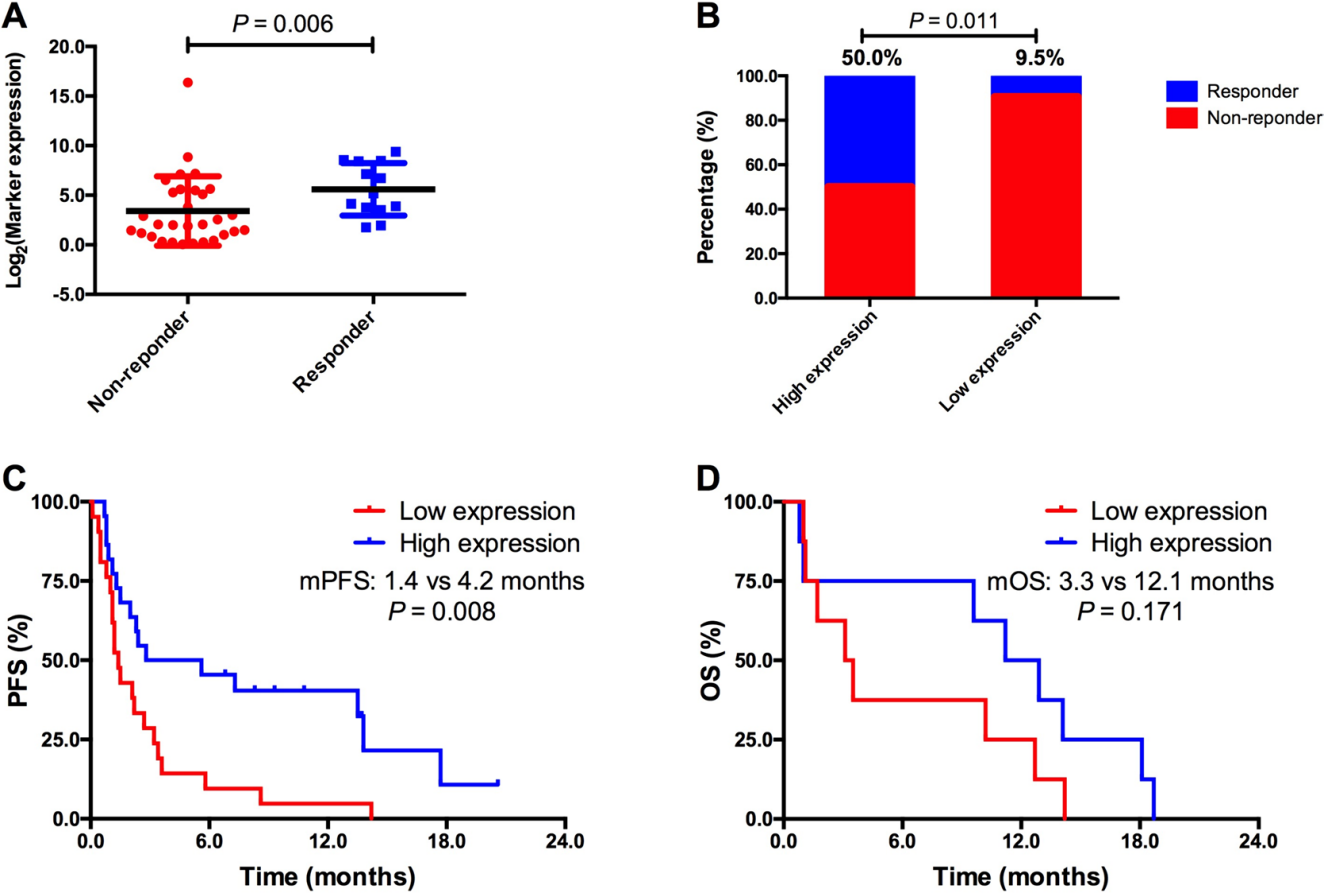
SIRPG expression was associated with response to PD-1 blockade in NSCLC. **A** SIRPG expression levels of Responders and Non-responders. Percentages of Responders and Non-responders (**B**), PFS (**C**), or OS (**D**) in SIRPG^high^ and SIRPG^low^ groups. PFS, Progression-free survival; OS, Overall survival

Generally, tumors with “hot” tumor immune phenotypes are likely to respond to immunotherapy. Having noticed the immune-responsive phenotype of NSCLC with high SIRPG expression, we next hypothesized that NSCLC patients whose pre-treatment tumors with high SIRPG expression may show good response to PD-1 blockade. To determine the impact of SIRPG expression on clinical outcomes in patients treated with PD-1 blockade monotherapy, we conducted an integrated analyses of the transcriptomic and treatment outcome data of patients with advanced NSCLC receiving PD-1 blockade monotherapy. Two publications with 43 NSCLC patients had available transcriptomic and response data [33, 34]. Firstly, we observed that responders had significantly higher SIRPG expression level than non-responders (*P* = 0.006, Fig. 5A). Patients with high SIRPG expression (≥ median level) showed a markedly increased response rate than those with low SIRPG expression (< median level) (ORR 50.0% vs. 9.5%, *P* = 0.011, Fig. 5B). Importantly, patients with high SIRPG expression had dramatically longer PFS than those with low SIRPG expression (median PFS 4.2 vs. 1.4 months, *P* = 0.008, Fig. 5C). OS was also longer in high SIRPG expression group than in low group though it did not reach the statistical significance, mainly due to the limited sample size (median OS 12.1 vs. 3.3 months, *P* = 0.171, Fig. 5D). We did not perform the multivariate analysis because the clinicopathological data of these studies were unavailable. To confirm the predictive value of SIRPG expression, we evaluated the association between SIRPG expression and treatment outcomes in 114 melanoma patients treated with anti-PD-1/PD-L1 monotherapy. The results showed that patients with high SIRPG expression had significantly prolonged OS than those with low SIRPG expression (Van Allen et al. cohort: *P* = 0.015; Gide TN et al. cohort: *P* = 0.011; Fig. S12) [35, 36].

### Distribution of SIRPG expression on distinct cell types

**Fig. 6:**
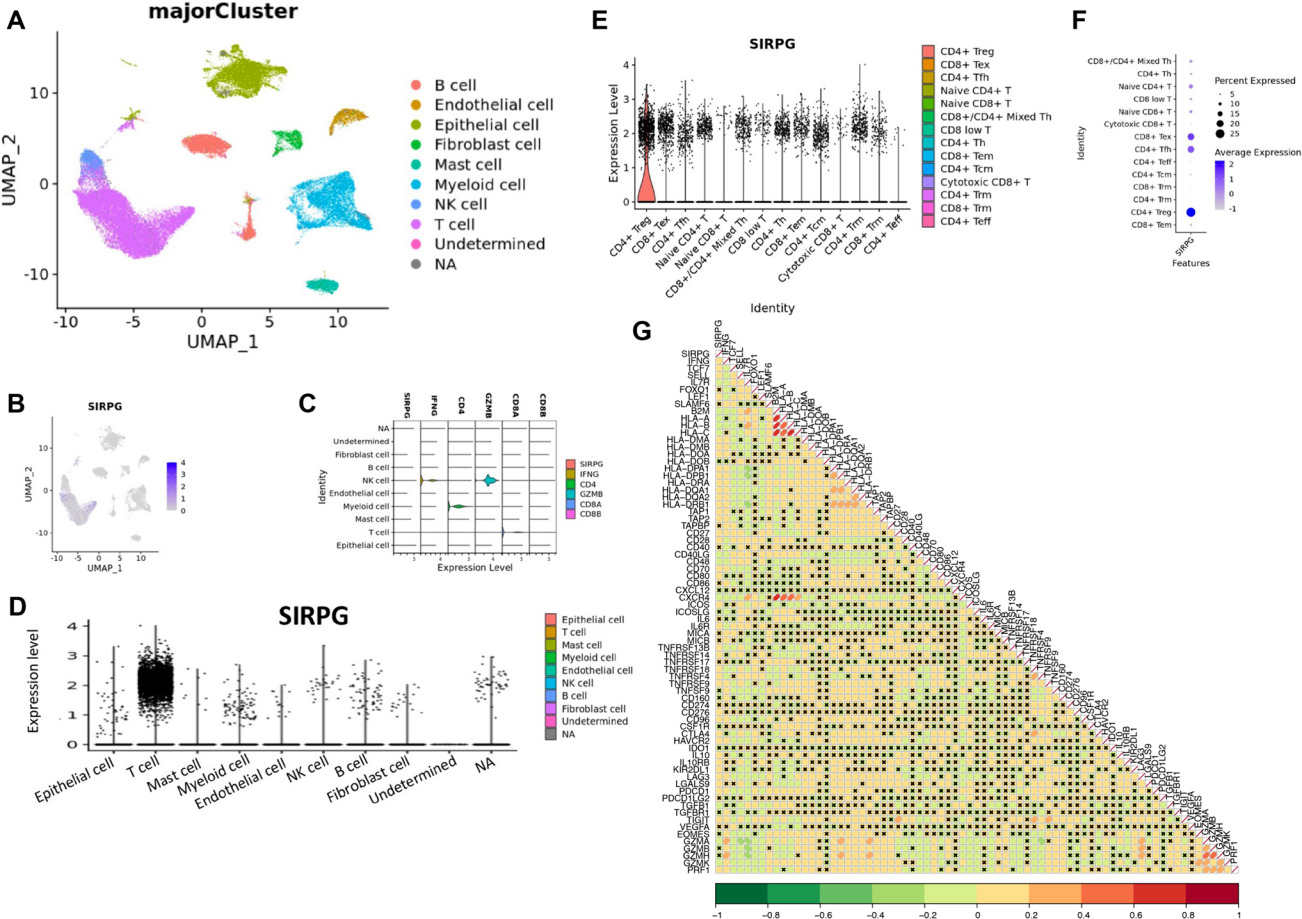
SIRPG expression by scRNA-seq. UMAP plots colored by clusters (**A**), SIRPG expression (**B**). CD8/CD4/GZMB/IFGN/SIRPG expression (**C**), SIRPG rank (**D**) among clusters. SIRPG rank (**E**), intensity (**F**) in T cell subtypes, and association with T cell-related genes in T cells (**G**). UMAP, Uniform manifold approximation and projection

To investigate the expression characteristics of SIRPG, we analyzed high-quality single-cell RNA sequencing data from 27 NSCLC patients. After data processing, a total of 103,599 cells were collected for analysis, including 38,101 T cells (Fig. 6A). Among all cell subtypes, we found that SIRPG was mainly expressed on T cells, followed by tumor cells (Fig. 6B–D). Our previous work studied the expression traits and regulatory mechanisms of SIRPG in lung tumor cells [22]. Here, we characterized SIRPG expression across 14 T cell subtypes by unsupervised graph-based clustering. As Fig. 6E–F suggested, CD8^+^ Tex and CD4^+^ Tregs showed the highest SIRPG expression.

Next, we focused on T cells and conducted correlation analysis to investigate whether SIRPG expression might affect the expression levels of MHC, immunostimulatory, immunoinhibitory, and cytotoxic molecules, respectively (Fig. 6G). Our analysis showed that SIRPG expression was significantly positively correlated with expressions of MHC molecules including HLA-DMA (*P* < 0.001), HLA-DMB (*P* < 0.001), HLA-DOA (*P* = 0.005), HLA-DPA1(*P* < 0.001), HLA-DPB1(*P* < 0.001), HLA-DRA (*P* = 0.002), HLA-DQA1 (*P* < 0.001), HLA-DQA2 (*P* < 0.001), HLA-DRB1 (*P* < 0.001), TAP1 (*P* < 0.001), TAP2 (*P* = 0.005), immunostimulatory molecules including CD27 (*P* < 0.001), CD28 (*P* < 0.001), CD70 (*P* < 0.001), CD80 (*P* < 0.001), ICOS (*P* < 0.001), IL6R (*P* < 0.001), TNFRSF13B (*P* < 0.001), TNFRSF14 (*P* < 0.001), TNFRSF18 (*P* < 0.001), TNFRSF4 (*P* < 0.001), TNFRSF9 (*P* < 0.001), immunoinhibitory molecules including PDCD1 (*P* < 0.001), LAG3 (*P* < 0.001), HAVCR2 (*P* < 0.001), CTLA4 (*P* < 0.001), TIGIT (*P* < 0.001), CD276 (*P* = 0.044), CD96 (*P* = 0.009), IL10 (*P* = 0.041), IL10RB (*P* < 0.001), KIR2DL1 (*P* = 0.004), LGALS9 (*P* < 0.001), and cytotoxic molecule IFNG (*P* = 0.001) in all T cells, while GZMA (*P* < 0.001), GZMK (*P* < 0.001), CXCR4 (*P* < 0.001), B2M (*P* < 0.001), HLA-A (*P* < 0.001), HLA-B (*P* < 0.001), HLA-C (*P* < 0.001), IL7R (*P* < 0.001), CD48 (*P* = 0.003), and CD40LG (*P* = 0.009) showed the negative association with SIRPG expression (Fig. 6G). For CD4^+^ Tregs and CD8^+^ Tex, SIRPG expression showed positive correlations with immune checkpoints including CTLA4, LAG3, PDCD1, TIGIT. No association was observed between SIRPG and IFNG expression in CD4^+^ Treg cells and weakly positive correlation in CD8^+^ Tex cells (Fig. S13).

### The biological effect of SIPRG expression in T cells

**Fig. 7:**
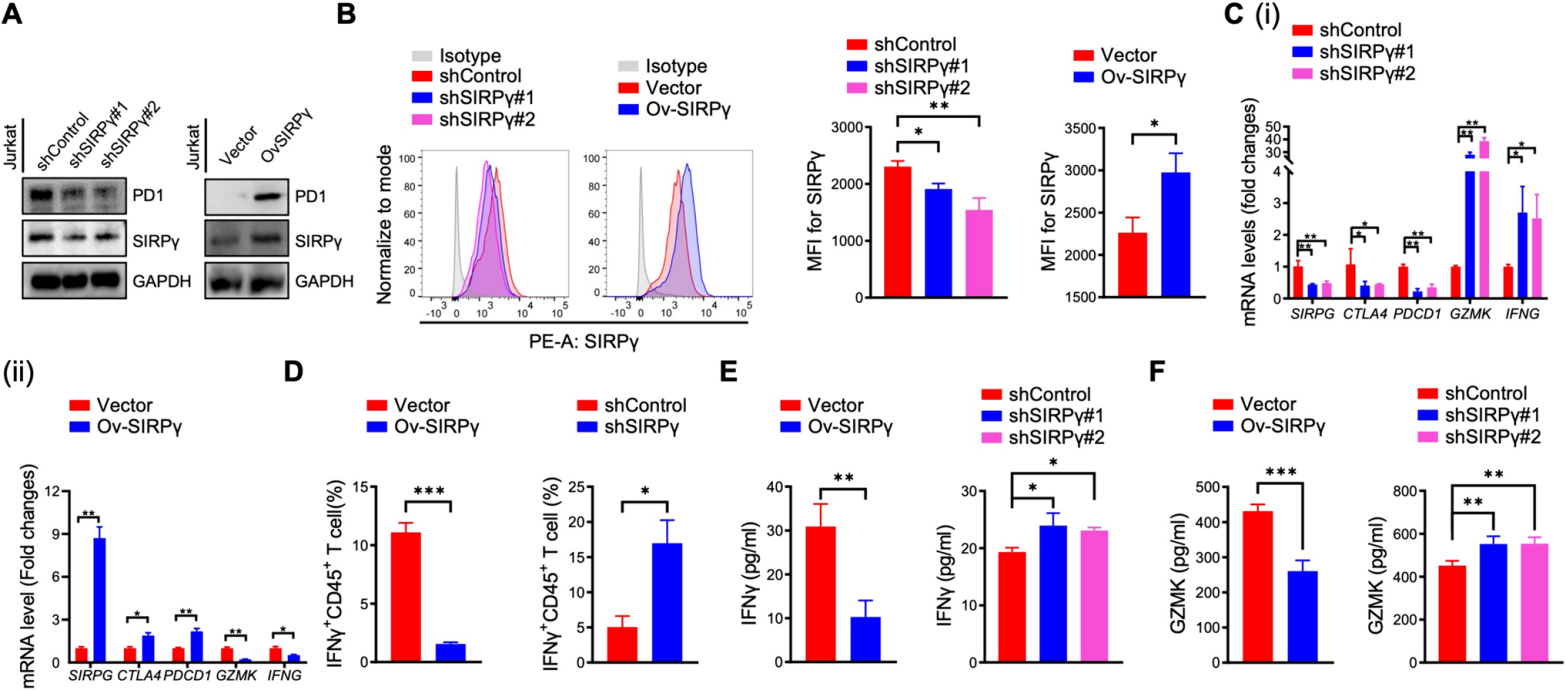
Biological effects of SIPRG in T cells. shSIRPG and ovSIRPG Jurkat cells confirmed by WB (**A**) or flow cytometry (**B**), and its association with immune-related molecule expression confirmed by qRT-PCR (**C**), flow cytometry (**D**), and ELISA (**E**, **F**). *P < 0.05; **P < 0.01; ***P < 0.001. MFI, Mean fluorescence intensity

Both the bulk RNA-seq and scRNA-seq data suggested the significant associations between SIRPG and immune checkpoints such as PDCD1, CTLA4, LAG3, and TIGIT, while the functional role of SIRPG expression in T cell mediated-adaptive antitumor immunity remains unknown. Whether SIRPG could regulate these immune checkpoints also remains undetermined. To further investigate the biological effect of SIRPG expression in T cells, we established SIRPG knockdown (shSIRPG) or overexpressed (ovSIRPG) Jurkat T cells in vitro (Figs. 7A, B and S14). qRT-PCR and western blot were conducted to detect the expression change of immune-related molecules in Jurkat T cells. The results showed that the expression of key immune-related molecules such as PDCD1 and CTLA4 were significantly increased in ovSIRPG Jurkat T cells compared with control groups, while cytotoxic molecules including IFNG and GZMK were significantly downregulated (Fig. 7A, C), while knockdown of SIRPG in Jurkat T cell showed the opposite effect (Fig. 7A, C). Flow cytometry and ELISA confirmed that IFNG and GZMK expression was significantly increased in shSIRPG Jurkat T cells compared with the control groups, which were backward verified in ovSIRPG Jurkat T cells (Fig. 7D–F). These results indicated that SIRPG expression in T cells could regulate the expression of key immune checkpoint molecules, resulting in the transition of T cell’s phenotype and cytotoxicity.

## Discussion

SIRPG is exclusively expressed in humans as well as primates, which binds the ubiquitous protein CD47 [42, 43]. Previous studies showed that it could interact with CD47 and plays an important role in T cell trans-endothelial migration, antigen-presenting related T cell proliferation and co-stimulation in specific conditions [44, 45]. However, the relationship between SIRPG expression and tumor microenvironment and whether SIRPG expression impacts response to immune checkpoint inhibitors remains elusive. To our knowledge, this study firstly and comprehensively investigated the associations between SIRPG expression with tumor immune microenvironment phenotypes, its functional role in shaping T cell mediated-adaptive antitumor immunity, as well as its predictive value for PD-1 blockade in various cancers. We integrated and analyzed scRNA-seq data, transcriptomic data and associated clinical outcomes from GEO, TCGA datasets and four clinical cohorts in patients treated with PD-1 blockade. The results showed that (i) most tumors had significantly higher level of SIRPG expression than matched normal tissues and high SIRPG expression correlated with a “hot” tumor immune phenotype; (ii) high SIRPG expression was associated with markedly better response to PD-1 blockade in both NSCLC and melanoma; (iii) SIRPG expression in T cells facilitated expression of several immune checkpoint molecules (e.g., PDCD1 and CTLA4), resulting in the transition of T cell’s phenotype and cytotoxicity. Taken together, these results suggested that SIRPG plays a significant role in T cell mediated-adaptive antitumor immunity and would be a promising predictive biomarker for PD-1 blockade in cancers and a novel immunotherapeutic target.

In this study, we systematically depicted the pan-cancer expression profiles of SIRPG among 33 cancer subtypes from TCGA database and characterized the relevance between SIRPG expression and tumor immune landscape. We demonstrated that SIRPG was overexpressed than paired normal tissues. SIRPG high-expressed tumors exhibited immunologically “hot” tumor immune microenvironment phenotype, with more tumor-infiltrating cells of both adaptive and innate immune systems such as T, B, NK cells, and DCs. Such alterations were observed in 29 of 33 cancer subtypes in our study, suggesting it a common phenomenon regardless of tumor histology. Tumors with high SIRPG expression also exhibited higher expression of genes regulating T cell activation and cytotoxicity. For example, expression of CD8A, GZMK, IFNG, and PRF1 was significantly increased in high SIRPG expression group. Pathway enrichment analysis of DEGs in NSCLC showed that high SIRPG expression was significantly enriched in pathways associated with T cell activation, cytokine–cytokine receptor interaction, antigen processing and presentation and so on. Consistent with these findings, previous studies reported SIRPG expressed in all naïve as well as central memory T cells, which could be upregulated when T cell activation [24, 42]. Meanwhile, anti-SIRPG or CD47 therapy could impair T cell activation [44]. In addition, by the blockade of the SIRPG/CD47 interaction, T cell interaction with CD47 was found important for both T cell migration and activation, especially under chronic stimulation conditions [24].

Based on the important roles of SIRPG in both tumor and immune cells, we evaluated the SIRPG-related expression traits, enrichment pathways, and immune infiltration characteristics in both LUAD and LUSC. We found that NSCLC with high SIRPG expression was significantly correlated with high expression levels of antigen presentation machinery, immunostimulatory, immunoinhibitory molecules, as well as high expression of genes regulating T cell activation and cytotoxicity, suggesting that lung tumors with high SIRPG expression had inflamed immune phenotypes. In NSCLC patients receiving PD-1 blockade, SIRPG is highly expressed in responders and patients with high levels of SIRPG expression were associated with better clinical outcomes, which was validated in the melanoma cohort. These results suggested that SIRPG expression might represent a potential biomarker to predict the response to PD-1 blockade in both NSCLC and melanoma. Further investigations with larger sample sizes are needed to validate its predictive value.

Our previous study investigated the expression level and biological functions of SIRPG in lung tumor cells [22]. Here, we mainly identified its expression characteristics in immune cells. Using scRNA-seq data from 27 lung cancer patients, we identified expression levels of SIRPG in different cell subtypes and found SIRPG was mainly expressed in T cells, especially CD8^+^ Tex and CD4^+^ Tregs. Correlation analysis suggested the positive correlation between SIRPG and several immune checkpoints such as PDCD1, CTLA4, LAG3, and TIGIT. Then, we studied the biological effect of SIRPG expression on Jurkat T cells via in vitro test and revealed SIRPG expression on T cells could facilitate expression of key immune checkpoint and cytotoxic molecules. These findings revealed that SIRPG would play a significant role in CD8^+^ T cell phenotype and function transition via regulating the expression of immune checkpoint and cytotoxic molecules. We observed a positive association between the expression of SIRPG and IFNγ or GZMK in human samples, whereas in vitro they showed a negative relationship. This phenomenon suggested that a hot tumor immune microenvironment might contribute to the upregulation expression of SIRPG, which in turn led to the exhaustion of T cells to avoid excessive immune activation. Unraveling the detailed mechanism of SIRPG regulating expression of immune checkpoint molecules and mediating CD8^+^ T cell phenotype and function transition is worthwhile for the development of novel immunotherapeutic targets and combination therapeutic strategies.

Collectively, SIRPG/CD47 interaction seems to display a complex role in tumors and their immune microenvironments. We previously observed that SIRPG-expressing lung cancer cells displayed stemness properties and transmitted the immune escape signal through sustaining CD47 expression. In this study, we found that high SIRPG expression was associated with an inflamed tumor immune microenvironment and a better response to PD-1 blockade. Notably, we revealed the significant role of SIRPG in regulating the expression of immune checkpoint and cytotoxic molecules. Totally, our results indicated that high SIRPG expression in the hot tumor immune microenvironment plays a vital role in restraining anti-tumor immunity. The high expression of SIRPG might be a consequence of a hot tumor microenvironment, which subsequently promoted the dysfunction of T cells to avoid excessive immune activation and attenuated anti-tumor immunity. Therefore, in terms of inhibiting tumor cells themselves and enhancing anti-tumor immunity, SIRPG serves as an important treatment target.

It is important to consider the following limitations when evaluating our findings. Firstly, the sample size of NSCLC patients receiving PD-1 blockade was relatively small, with only 43 patients. Further investigation with a larger patient population is needed to validate our findings. Secondly, the interplay between SIRPG expression and the tumor microenvironment was not fully elucidated and warrants additional exploration.

In conclusion, the current study reported that SIRPG expression positively correlated with an “hot” tumor immune phenotype and favorable response to PD-1 blockade. Functionally, SIRPG could regulate expression of immune checkpoint molecules to mediate T cell phenotype and function transition. Our study suggests that SIRPG would be a promising predictive biomarker for PD-1 blockade and novel immunotherapeutic target in cancers.

### Supplementary Information

Below is the link to the electronic supplementary material.

Supplementary file1 (DOCX 38185 KB)

## Data Availability

Data are available upon reasonable request. The data used to support the findings of this study are available from the corresponding author upon request.

## Abbreviations

B2M: β2-Microglobulin
CI: Confidence interval
COAD: Colon adenocarcinoma
CR: Complete response
DCs: Dendritic cells
DEGs: Differentially expressed genes
ESCA: Esophageal carcinoma
FDR: False discovery rates
GBM: Glioblastoma multiforme
GEO: Gene expression omnibus
GGN: Ground glass nodule
HNSC: Head and neck squamous cell carcinoma
HR: Hazard ratio
KIRC: Kidney renal clear cell carcinoma
KIRP: Kidney renal papillary cell carcinoma
LUAD: Lung adenocarcinoma
LUSC: Lung squamous cell carcinoma
MDSC: Myeloid-derived suppressor cell
MHC: Major histocompatibility complex
MSI: Microsatellite instability
NSCLC: Non-small-cell lung cancer
ORR: Objective response rate
OS: Overall survival
PCA: Principal component analysis
PD: Progressive disease
PFS: Progression-free survival
PR: Partial response
qRT-PCR: Quantitative real-time polymerase chain reaction
READ: Rectum adenocarcinoma
scRNA-seq: Single-cell RNA sequencing
SD: Stable disease
SIRPG: Signal regulatory protein gamma
SKCM: Skin cutaneous melanoma
STAD: Stomach adenocarcinoma
TCGA: The Cancer Genome Atlas
TMB: Tumor mutational burden
tSNE: T-distributed stochastic neighbor embedding
UCEC: Uterine corpus endometrial carcinoma
