# Supplementary material for: SIRPG expression positively associates with an inflamed tumor microenvironment and response to PD-1 blockade
Source: Cancer Immunol Immunother. 2024 Jun 4;73(8):147. doi: 10.1007/s00262-024-03737-y (PMC11150346; doi:10.1007/s00262-024-03737-y)
Supplement: Supplementary file 1 — Supplementary file1 (DOCX 38185 KB) [file 262_2024_3737_MOESM1_ESM.docx]

**Supplemental materials**

**SIRPG expression positively associates with an inflamed tumor microenvironment and response to PD-1 blockade**

Libo Luo, Minlin Jiang, Hong Wu, Yiqiang Liu, Haowei Wang, Caicun Zhou, Shengxiang Ren, Xiaoxia Chen, Tao Jiang, Chuan Xu

**Supplemental Figure S1……………………………..……………………………………………..2**

**Supplemental Figure S2……………………………..……………………………………………..3**

**Supplemental Figure S3……………………………..……………………………………………..4**

**Supplemental Figure S4……………………………..……………………………………………..5**

**Supplemental Figure S5……………………………..……………………………………………..6**

**Supplemental Figure S6……………………………..……………………………………………..7**

**Supplemental Figure S7……………………………..……………………………………………..8**

**Supplemental Figure S8……………………………..……………………………………………..9**

**Supplemental Figure S9……………………………..……………………………………………..10**

**Supplemental Figure S10……………………………..……………………………………………11**

**Supplemental Figure S11……………………………..……………………………………………12**

**Supplemental Figure S12……………………………..……………………………………………13**

**Supplemental Figure S13……………………………..……………………………………………14**

**Supplemental Figure S14……………………………..……………………………………………15**

**Supplemental Table S1………………………………..……………………………………………16**

**
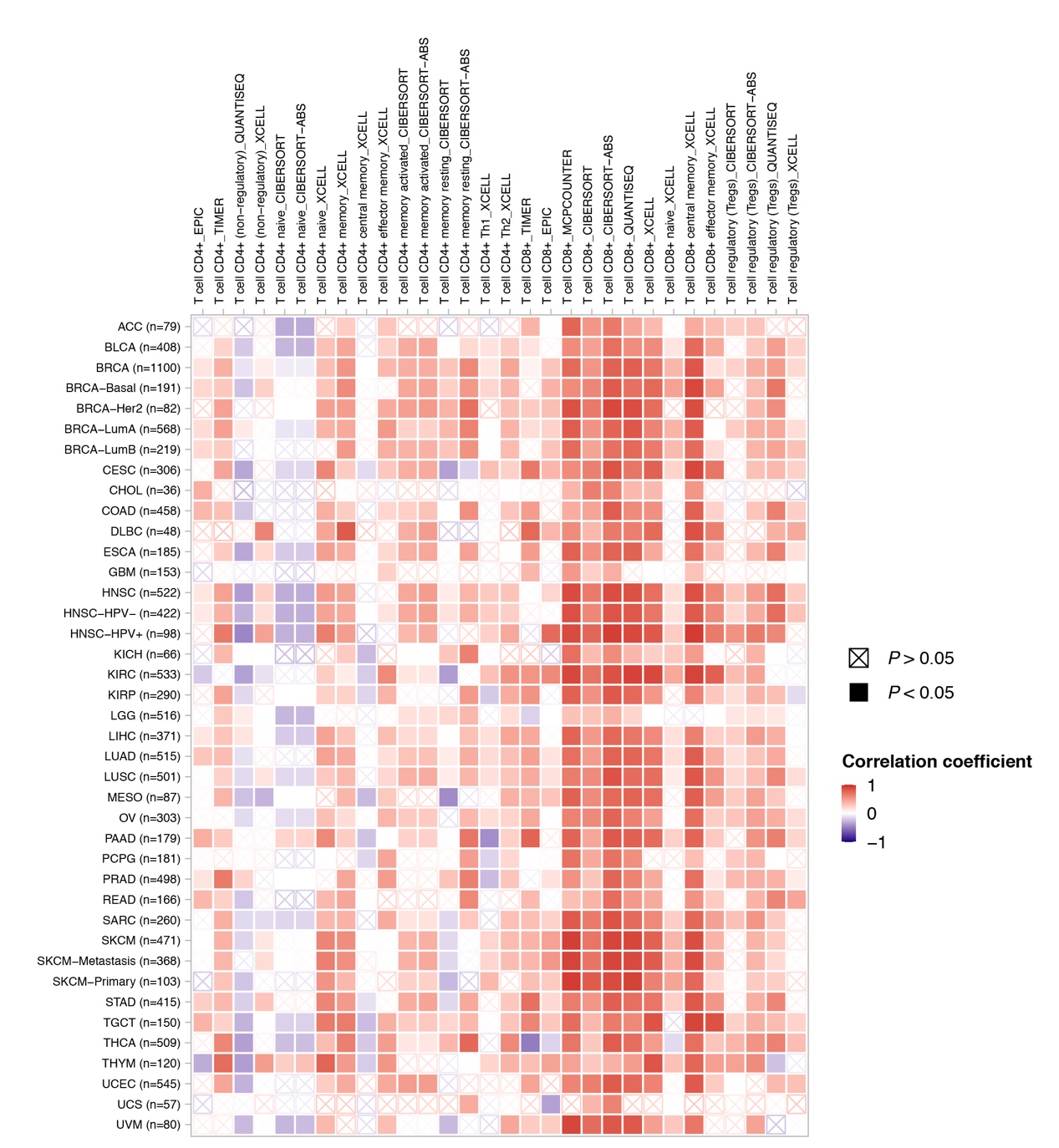
**

**Supplemental Figure S1.** The correlation analysis of SIRPG expression with distinct T cell subtypes among various cancers.


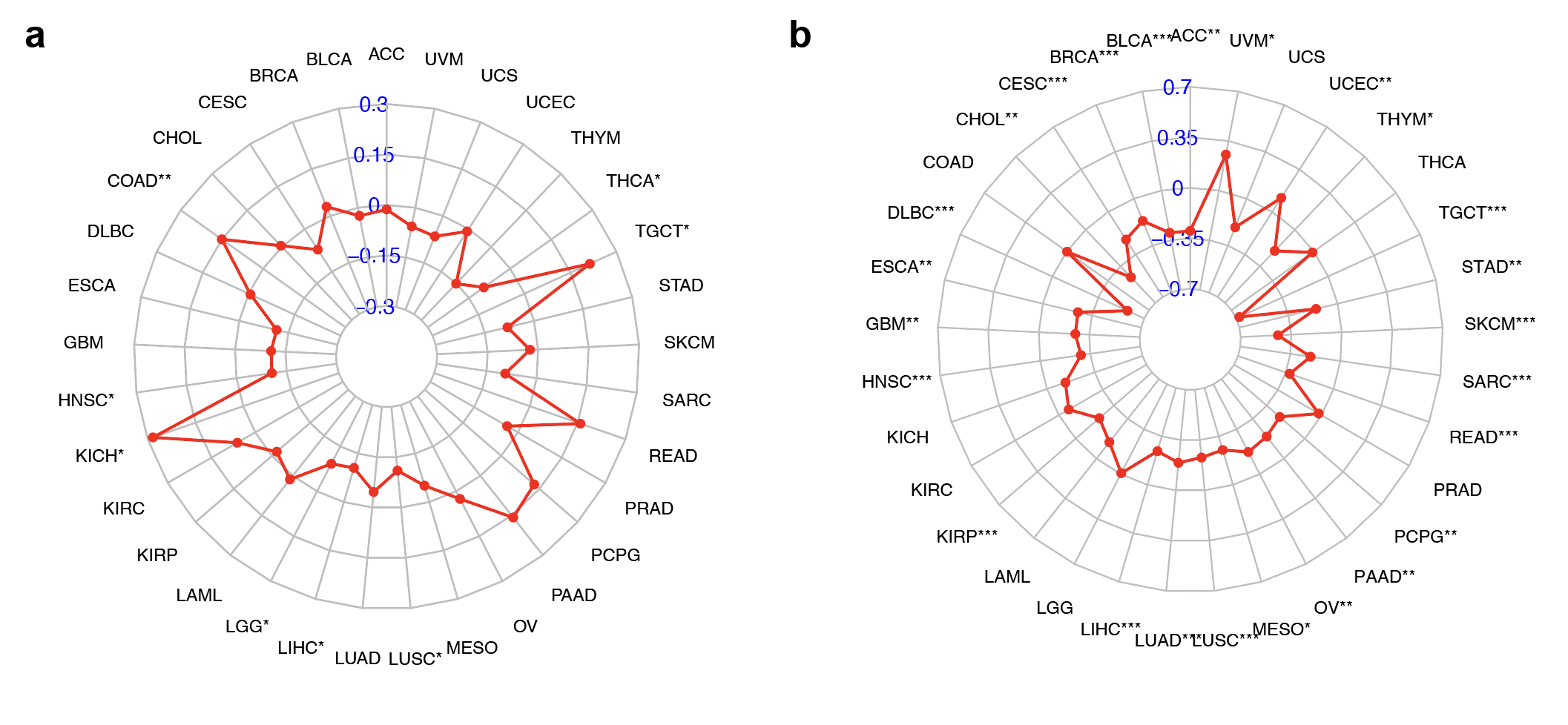


**Supplemental Figure S2.** Radar plots depicting the associations between SIRPG expression and TMB (a) or MSI (b). *P<0.05; **P<0.01; ***P<0.001.


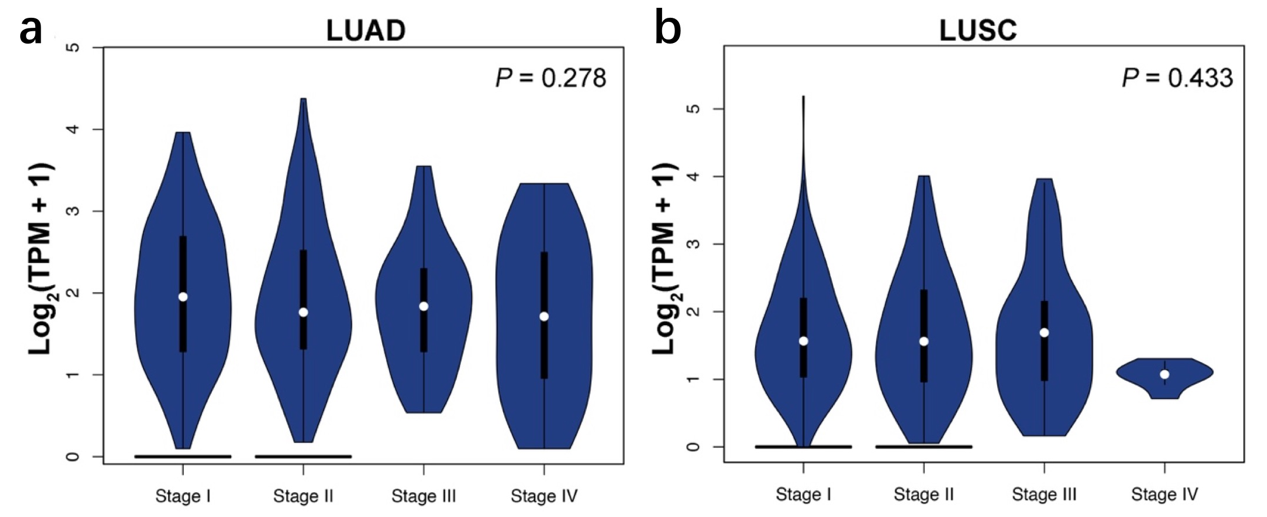
**Supplemental Figure S3**. The expression level of SIRPG across different stages in LUAD (a) and LUSC (b). TPM, Transcripts per kilobase million; LUAD, Lung adenocarcinoma; LUSC, Lung squamous cell carcinoma.


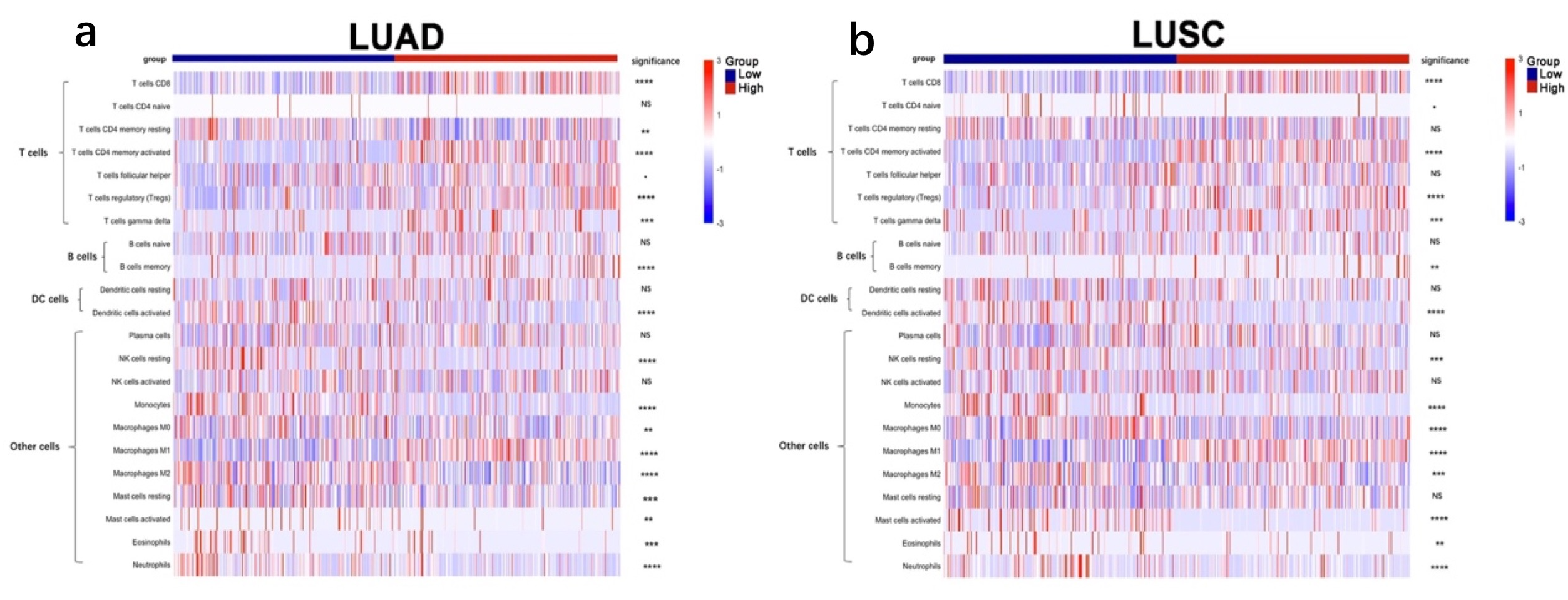


**Supplemental Figure S4**. The immune landscape between high and low SIRPG expression groups in LUAD (a) and LUSC (b) by CIBERSORT. LUAD, Lung adenocarcinoma; LUSC, Lung squamous cell carcinoma.


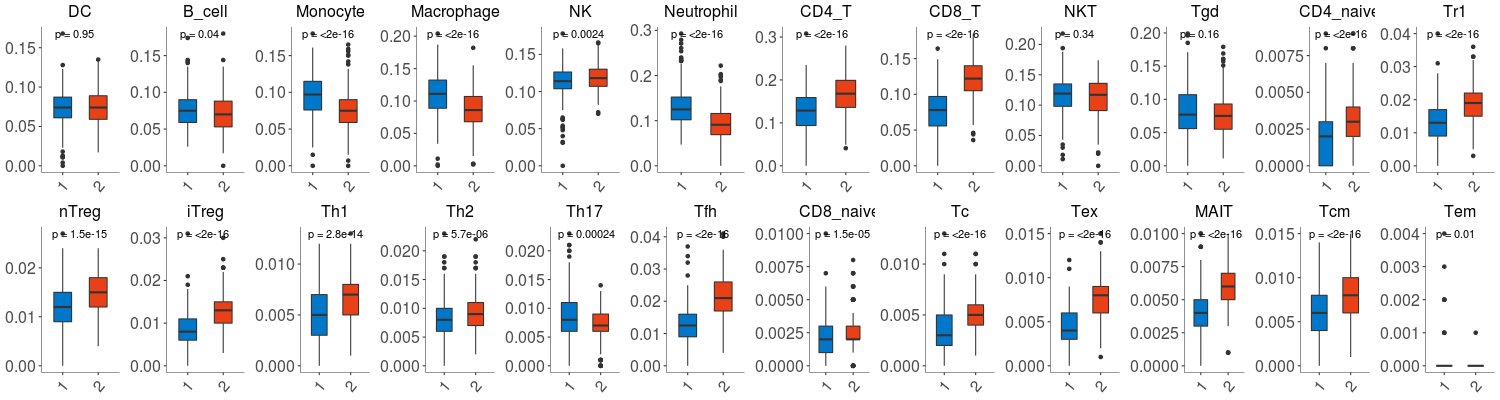


**Supplemental Figure S5**. The immune cell abundance between high and low SIRPG expression groups in LUAD calculated by ImmuCellAI.


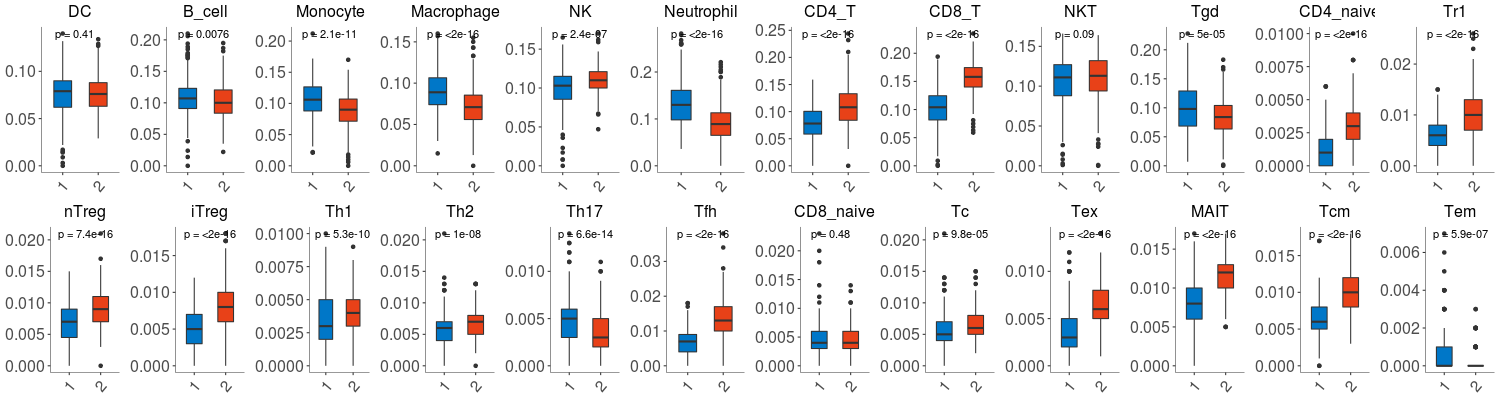


**Supplemental Figure S6**. The immune cell abundance between high and low SIRPG expression groups in LUSC calculated by ImmuCellAI.


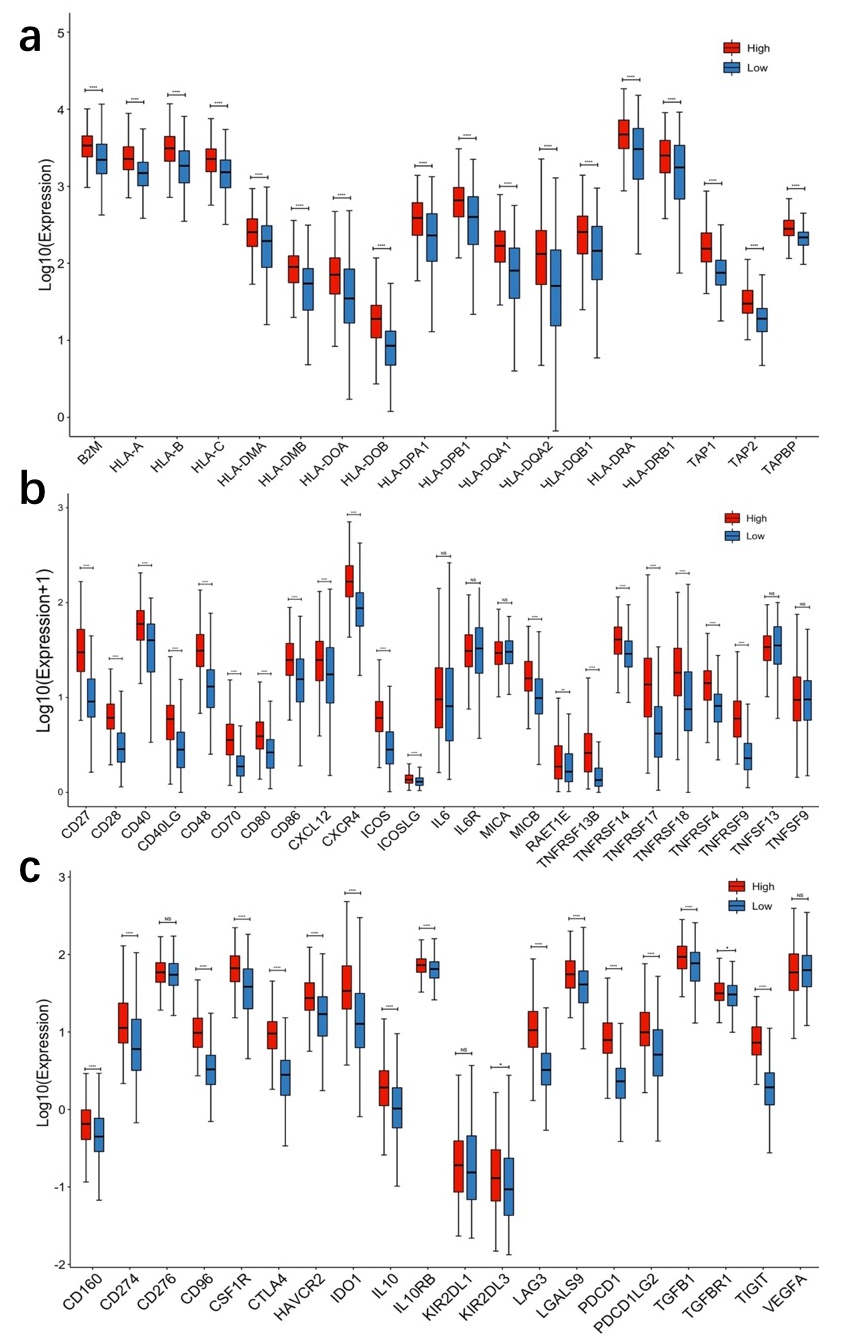


**Supplemental Figure S7**. The expression level of antigen presentation machinery [β2-microglobulin (B2M), HLA-A, HLA-B, HLA-C, HLA-DRB1, HLA-DRB5, TAP1, TAP2, TAPBP], immunostimulatory (CD28, CD40, CD80, CD86, ICOS), and immunoinhibitory (CD274, PDCD1, CTLA4, LAG3, TIGIT, HAVCR2) molecules between high and low SIRPG expression groups in LUAD. LUAD, Lung adenocarcinoma.


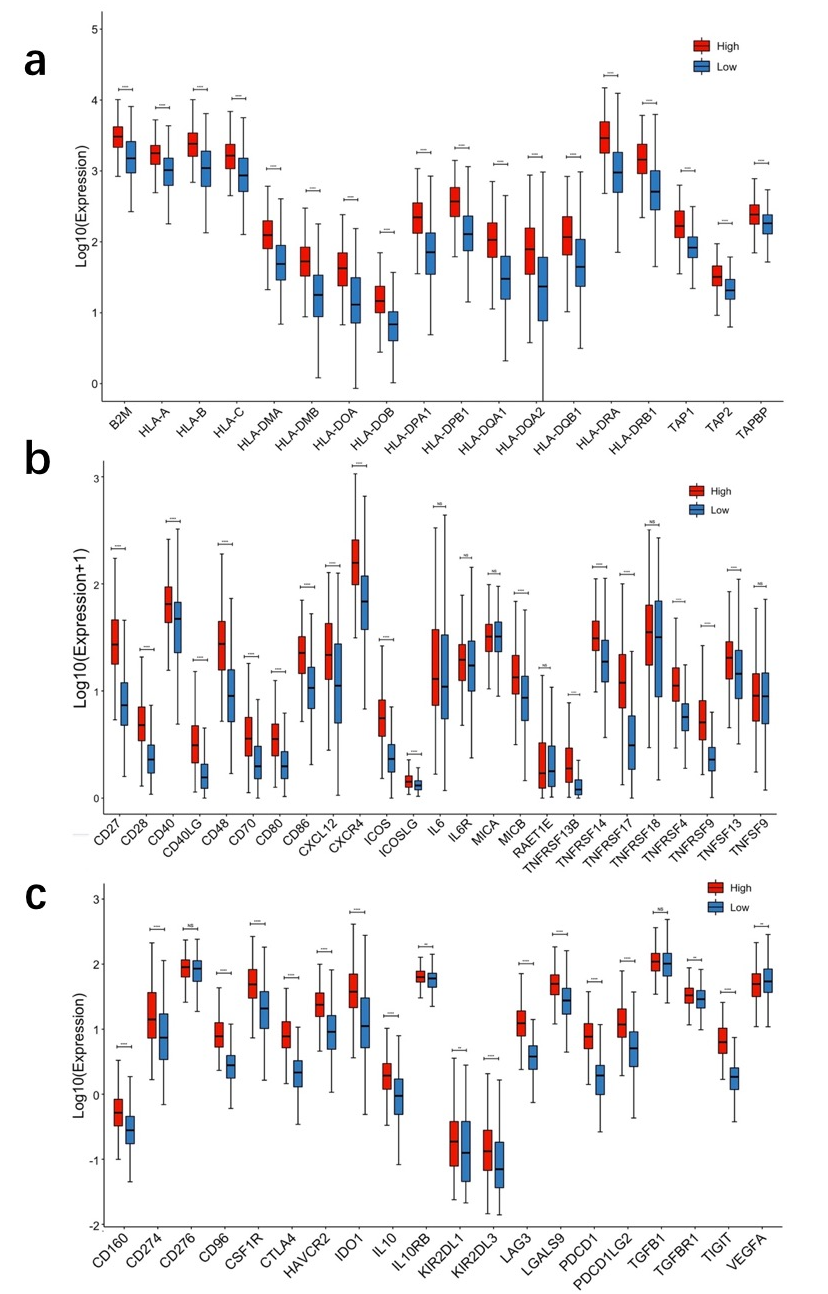


**Supplemental Figure S8**. The expression level of antigen presentation machinery [β2-microglobulin (B2M), HLA-A, HLA-B, HLA-C, HLA-DRB1, HLA-DRB5, TAP1, TAP2, TAPBP], immunostimulatory (CD28, CD40, CD80, CD86, ICOS), and immunoinhibitory (CD274, PDCD1, CTLA4, LAG3, TIGIT, HAVCR2) molecules between high and low SIRPG expression groups in LUSC. LUSC, Lung squamous cell carcinoma.


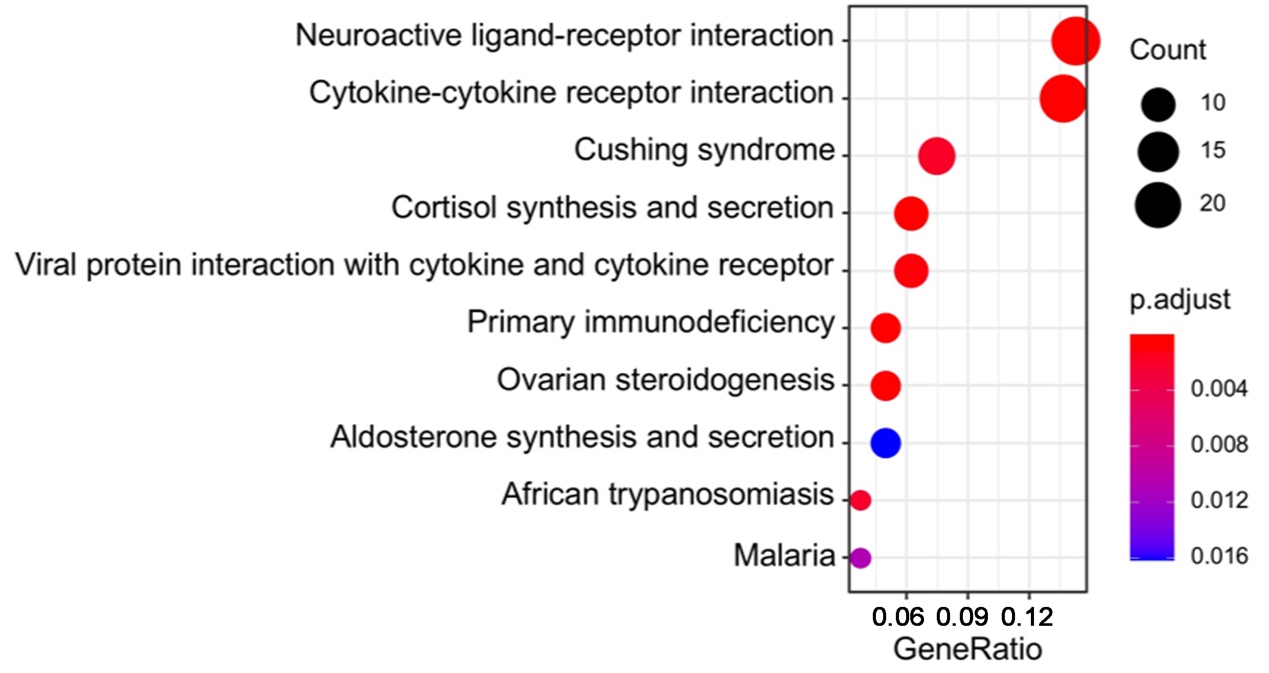


**Supplemental Figure S9**. Kyoto encyclopedia of genes and genomes (KEGG) pathway analysis of DEGs between high and low SIRPG expression groups in LUAD. LUAD, Lung adenocarcinoma.


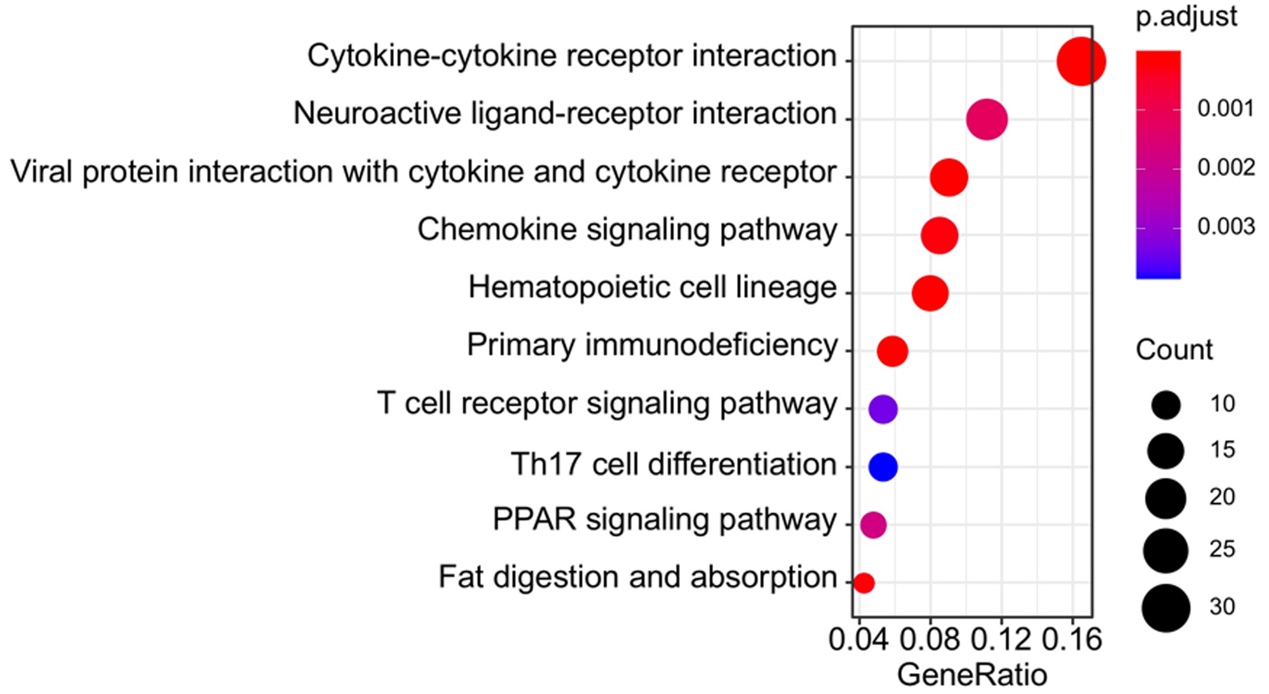


**Supplemental Figure S10**. Kyoto encyclopedia of genes and genomes (KEGG) pathway analysis of DEGs between high and low SIRPG expression groups in LUSC. LUSC, Lung squamous cell carcinoma.


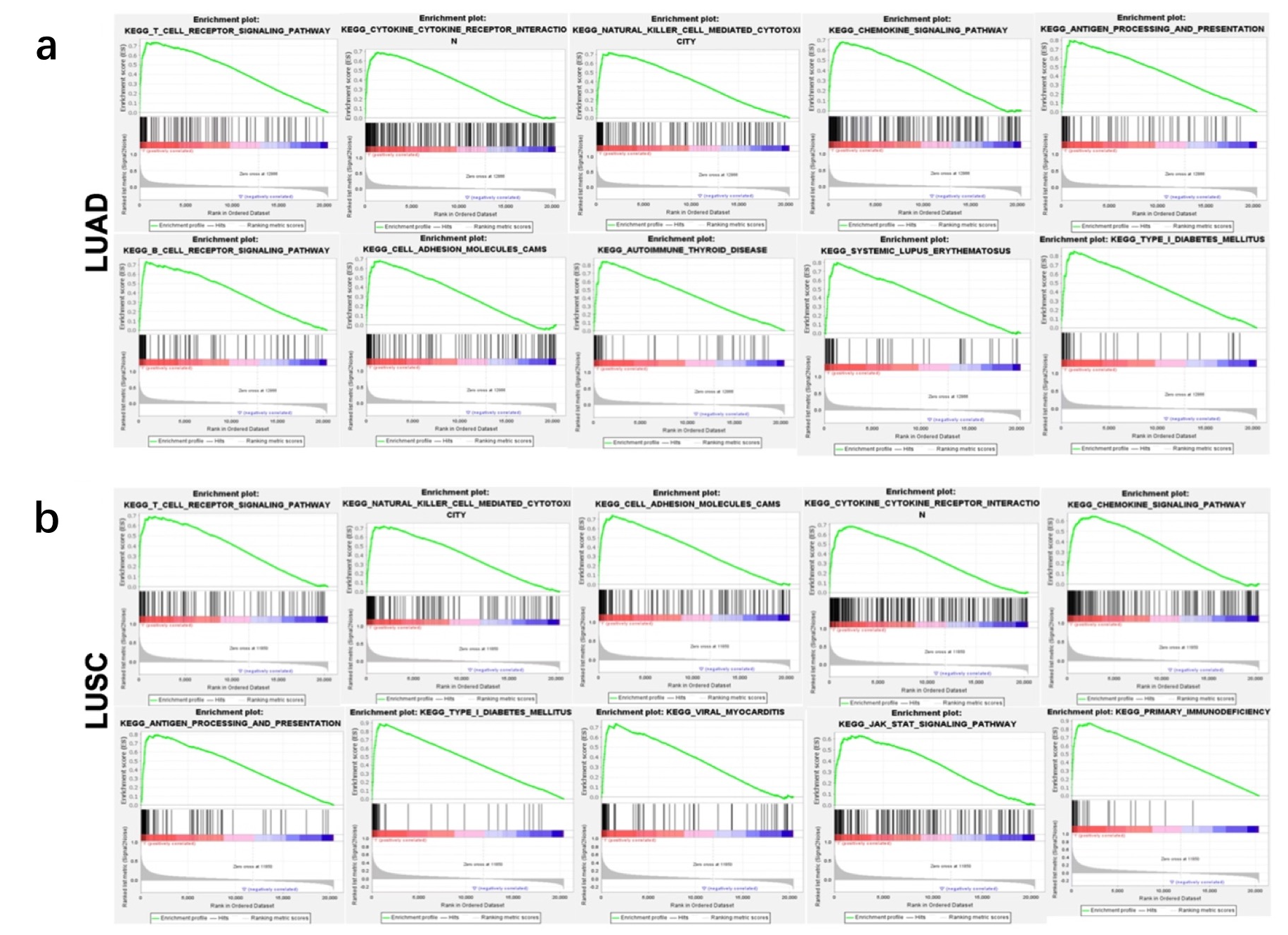


**Supplemental Figure S11**. GSEA analysis of the top ten significantly enriched pathways in LUAD (a) and LUSC (b). LUAD, Lung adenocarcinoma; LUSC, Lung squamous cell carcinoma.


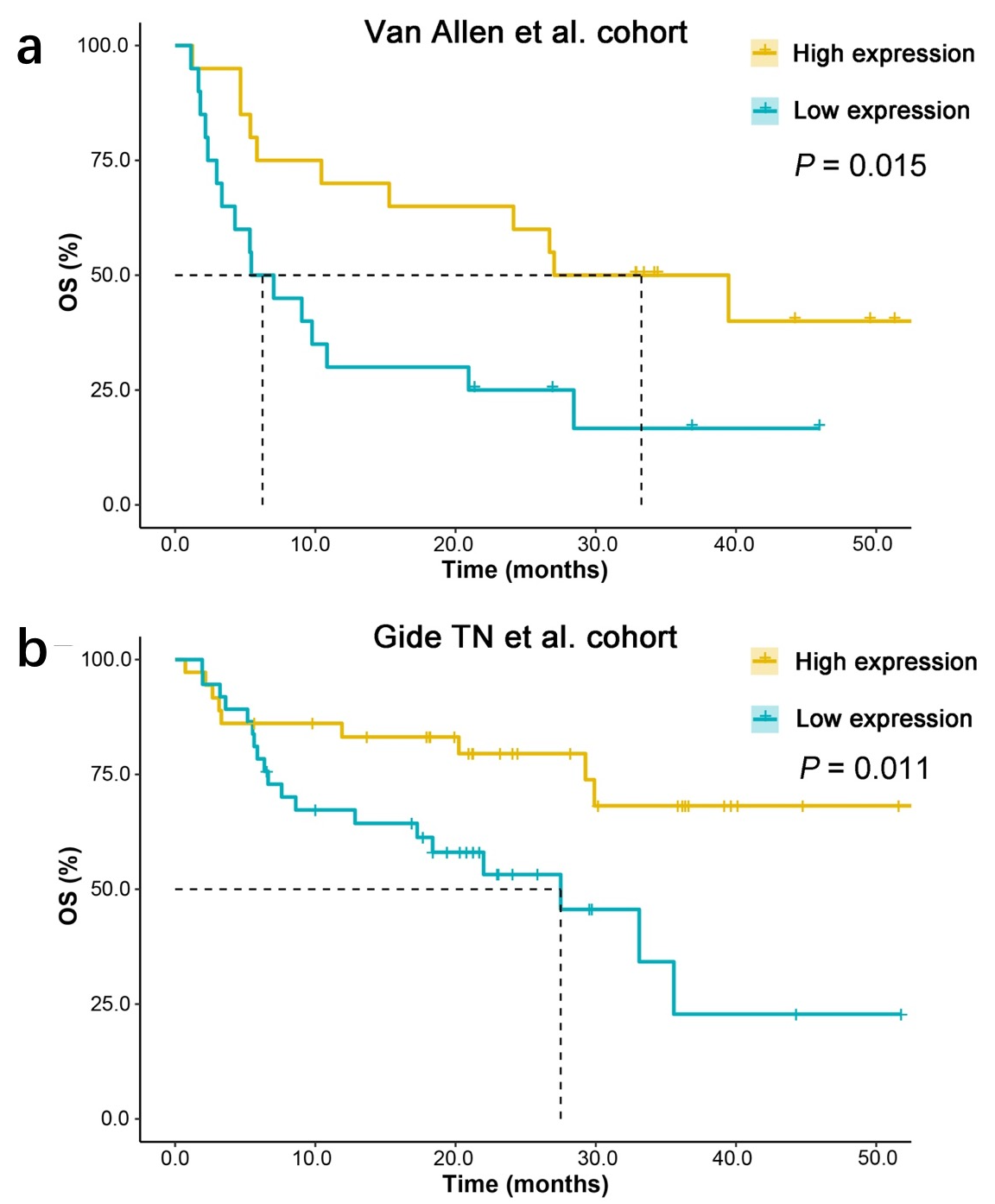


**Supplemental Figure S12**. SIRPG expression associated with response to PD-1/PD-L1 blockade in melanoma patients. a-b. Survival analysis of SIRPG expression in NSCLC patients with PD-1 blockade in Van Allen et al. cohort (a) and Gide TN et al. cohort (b). OS, Overall survival.

**
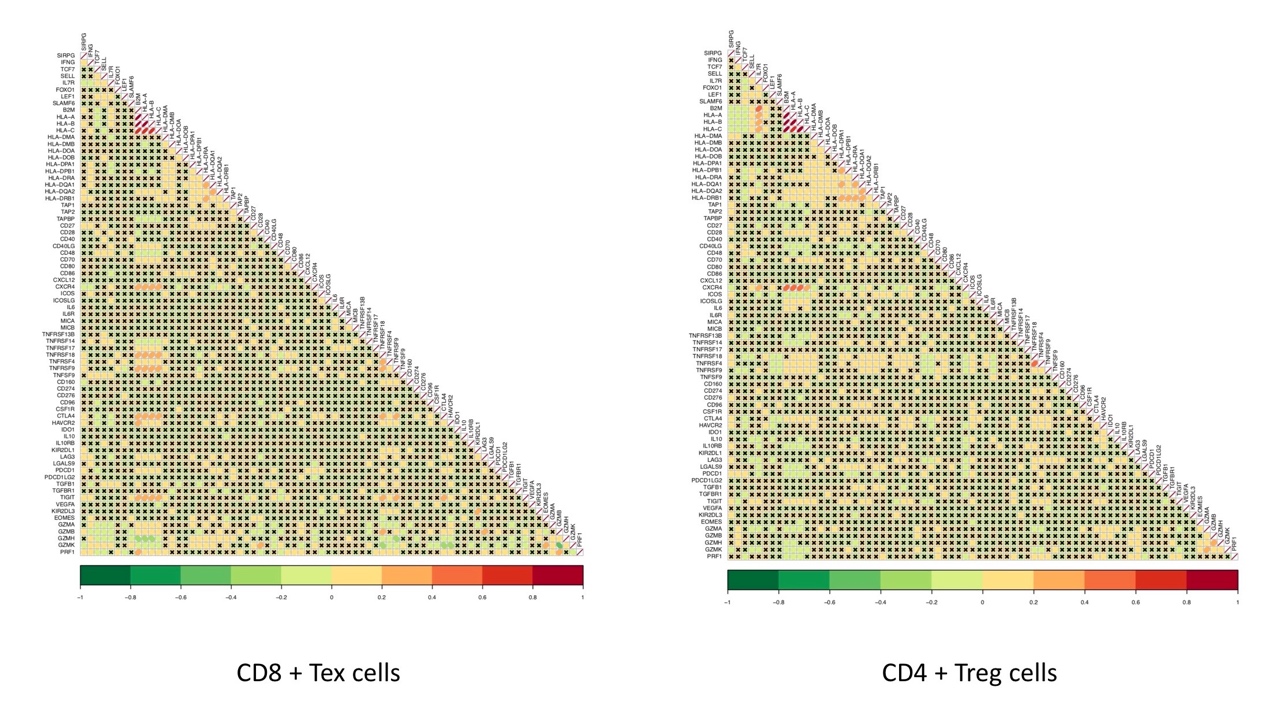
**

**Supplemental Figure S13**. Correlation analysis of SIRPG, major histocompatibility complex, immunostimulatory, immunoinhibitory and cytotoxic molecules expression level in CD8+ Tex cells and CD4+ Tregs.

**
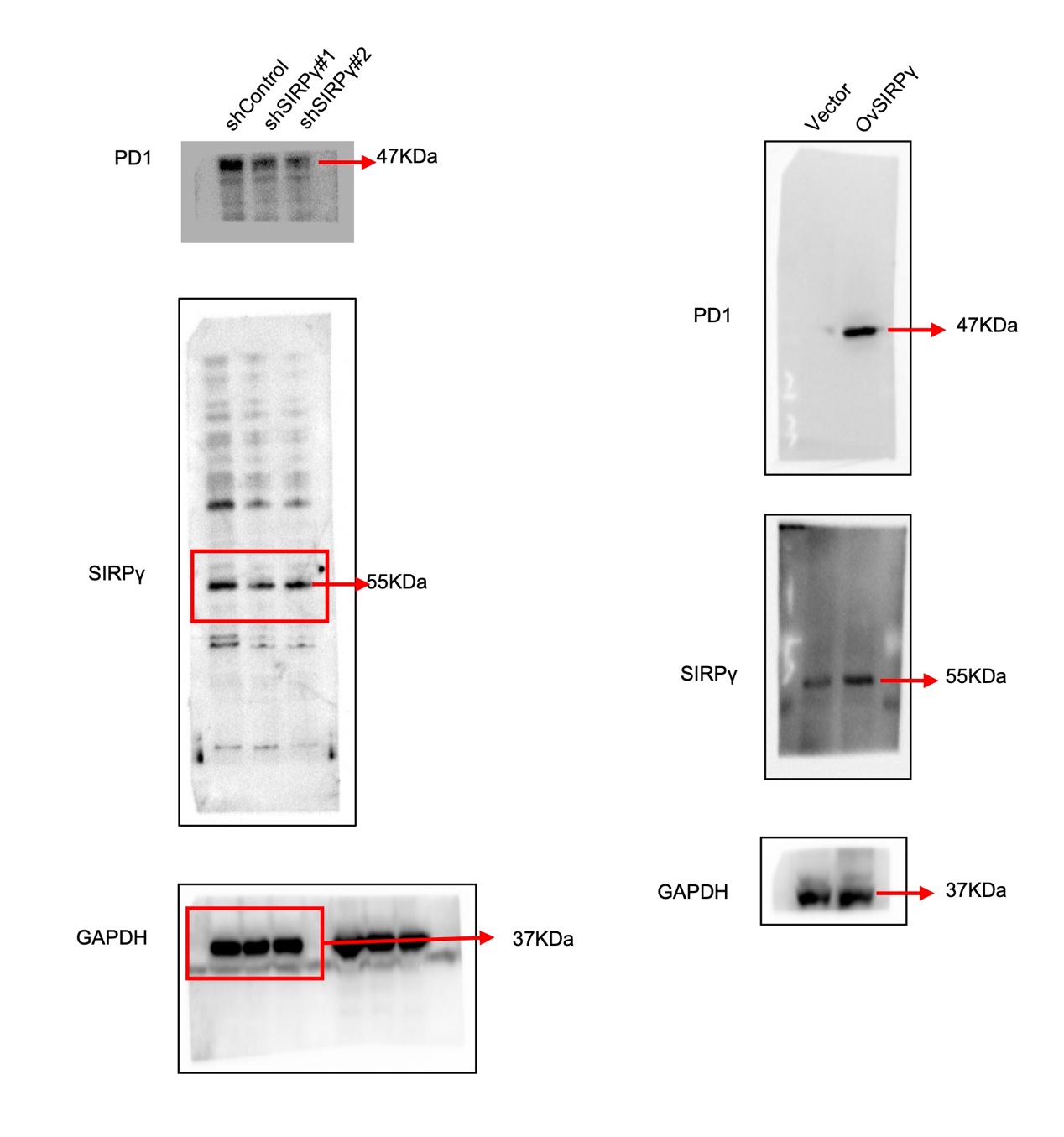
**

**Supplemental Figure S14**. Uncropped WB images for figure 7A.

**Supplemental Table S1. Sequences of the primers used for qRT-PCR.**

| Gene | Primer sequence (5’→3’) | Amplification  size (bp) |
| --- | --- | --- |
| IFNγ | F: AGCTCTGCATCGTTTTGGGTT  R: GTTCCATTATCCGCTACATCTGAA | 118 |
| PD1 | F: CAGTTCCAAACCCTGGTGGT  R: GGCTCCTATTGTCCCTCGTG | 114 |
| SIRPG | F: TCCTCCTGGTCCTTTCCT R: GGCTGTCTTTCCAACTGTG | 121 |
| GZMK | F: CGTTTGTGGAGGTGTTCTG  R: GAGAGAGTGTGCGCCTAAA | 112 |
| CTLA4 | F: GCAGTTAGTTCGGGGTTG  R: CATTCTGGCTCTGTTGGG | 128 |
| β-actin | F: TCTCCCAAGTCCACACAGG  R: GGCACGAAGGCTCATCA | 127 |
